# Flux Balance Analysis Inspired Bioprocess Upgrading for Lycopene Production by a Metabolically Engineered Strain of *Yarrowia lipolytica*

**DOI:** 10.3390/metabo5040794

**Published:** 2015-12-21

**Authors:** Komi Nambou, Xingxing Jian, Xinkai Zhang, Liujing Wei, Jiajia Lou, Catherine Madzak, Qiang Hua

**Affiliations:** 1 State Key Laboratory of Bioreactor Engineering, East China University of Science and Technology, 130 Meilong Road, Shanghai 200237, China; E-Mails: noelnambou@yahoo.fr (K.N.); Y10150130@mail.ecust.edu.cn (X.J.); 010140069@mail.ecust.edu.cn (X.Z.); weiliujing98@163.com (L.W.); rene2514@163.com (J.L.); 2 INRA, UMR1319 Micalis, Domaine de Vilvert, F-78352 Jouy-en-Josas, France; E-Mail: madzak@platon.grignon.inra.fr; 3 Shanghai Collaborative Innovation Center for Biomanufacturing Technology (SCICBT), 130 Meilong Road, Shanghai 200237, China

**Keywords:** *Yarrowia lipolytica*, lycopene, flux balance analysis, fermentation, metabolic network model yli v1.7

## Abstract

Genome-scale metabolic models embody a significant advantage of systems biology since their applications as metabolic flux simulation models enable predictions for the production of industrially-interesting metabolites. The biotechnological production of lycopene from *Yarrowia lipolytica* is an emerging scope that has not been fully scrutinized, especially for what concerns cultivation conditions of newly generated engineered strains. In this study, by combining flux balance analysis (FBA) and Plackett-Burman design, we screened chemicals for lycopene production from a metabolically engineered strain of *Y. lipolytica*. Lycopene concentrations of 126 and 242 mg/L were achieved correspondingly from the FBA-independent and the FBA-assisted designed media in fed-batch cultivation mode. Transcriptional studies revealed upregulations of heterologous genes in media designed according to FBA, thus implying the efficiency of model predictions. Our study will potentially support upgraded lycopene and other terpenoids production from existing or prospect bioengineered strains of *Y. lipolytica* and/or closely related yeast species.

## 1. Introduction

Carotenoids represent a subcategory of tetraterpenoid pigmented lipid compounds made by plants and various fungi and bacteria [1]. Majorly exemplified by ß-carotene, lycopene, astaxanthin, zeaxanthin, fucoxanthin, ß-cryptoxanthin, canthaxanthin, lutein, and crocetin, compounds of the carotenoids family handle a diversity of bright colors such as yellow, red, purple, and orange [2,3]. Lycopene is a bioactive phytochemical of this vast family of carotenoids that has gained much concerns these recent years because of its commercial attributes and due to its biofunctionality [4]. Exclusively synthesized on one hand, by plants like tomato, watermelon, guava, papaya, apricots, pink grapefruits, and red oranges and on the other hand by carotenogenic microorganisms, lycopene is essential for the human body given that it can only be provided through its ingestion under multiple forms such as food or drugs. Lycopene promotes good health owing to its therapeutic, prophylactic, and nutraceutical functions. Increasing scientific evidence displays its implications in carcinogenic cell apoptosis and cell cycle arrest of diverse types of human cancers [5,6,7,8]. Lycopene is also recognized as a strong cancer chemo-preventive agent [9,10] and has been found to have cardio-protective, antioxidant, and anti-inflammatory effects [11]. In view of the above, the large scale production of lycopene is of fundamental necessity. However, the current manufacture of lycopene from plant sources faces food competition, environment, high production cost, long processing time, and low yield problems. Besides, microbial fermentation using carotenogenic microorganism is scarce and fails to produce sizable titers of lycopene. Big efforts have been made to engineer non-carotenogenic hosts for lycopene production; but the fermentation process still constitutes the foremost impediment for generating great amounts of lycopene. *Yarrowia lipolytica*, categorized as GRAS (Generally Recognized As Safe), has been established as a promising fermentation platform. Recently, a complete procedure for engineering *Y. lipolytica* for lycopene production was developed. Nonetheless, apart from the culture medium and genetic manipulation procedure provided [12], no deep investigation of cultural conditions was communicated. In addition, a fed-batch process for lycopene production from this species was established, but authors did not focus on the improvement of the bioprocess route [13]. Furthermore, up to now, only one patent has reported lycopene production from engineered strains of *Y. lipolytica* under lipid accumulation conditions [14]. Therefore, endeavors need to be made for building complete innovative, cost-effective, and competitive bioprocesses for lycopene production from genetically engineered strains of *Y. lipolytica*. One of the most efficient tools for yielding high titers of carotenoids in engineered strains is based on knock-out or overexpression of specific genes coupled with computational approaches [15,16,17]. Moreover, whole-genome sequencing and high-throughput omics as well as the detailed biochemical and enzymatic documentation on microbial metabolism have promoted prosperous genome-scale metabolic network (GSMN) reconstruction [18,19].

*In silico* models are valuable for comprehending the biochemical aptitudes of an organism and to foresee the influence of genetic and/or medium perturbations on growth and metabolic fluxes [20,21]. Flux balance analysis (FBA) has been materialized as an operational *in silico* channel for scrutinizing biological networks in a quantitative approach. Contrary to mechanistic simulations that rely on precise kinetic information, FBA is based on the stoichiometric matrix and a biologically important objective function for identifying optimal reaction flux distributions which are subsequently used to unravel the metabolic capabilities of the studied system [22].

The present-day FBA is in a wide way to guide designs of experiments. In a previous study, we constructed a genome scale metabolic model of *Y. lipolytica* for lipid production and predicted a minimal culture medium for lipids biosynthesis. Afterward, we have updated this model for generating model information for terpenoids production. The updated model is apt to provide reliable information when farnesyl diphosphate (FPP), one of the key precursors of terpenoids backbone in *Y. lipolytica*, is resolved as the objective metabolite. We hypothesized that such a metabolic network model can support the prediction of culture media for ameliorating the yield of lycopene in biotechnological applications.

Statistical design methods, especially the Plackett-Burman design, are commonly employed for optimization of fermentation media. The Plackett-Burman designs are usually resolution III, highly efficient two level fractional factorial screening designs for studying N-1 variables using N runs, where N is a multiple of 4. They use the minimum number of runs to quickly identify the factors with a significant effect on the response. However, the choice of the initial set of chemical compounds is laborious since these compounds are generally randomly selected. In addition, though GSMN can predict the nutritional requirements for a given biological system, the accuracy of the amount of a single predicted compound to be added is difficult to determine because *in silico* data rarely match with the in vivo metabolic behavior. However, by combining GSMN (FBA) results and statistical methods such as Plackett-Burman design, it is possible to develop an efficient culture medium for the production of valuable objective products.

Therefore, in this paper, we run FBA by means of COBRA Toolbox 2.0 [23] to foresee environmental conditions contributing to FPP overproduction that may well redirect the carbon flux toward lycopene biosynthesis in an engineered strain of *Y. lipolytica* harboring only *crtE*, *crtB*, and *crtI* genes. As model predictions do not correctly reflect the in vivo situation, we combined modelling data with Plackett-Burman factorial design of experiments for optimizing cultivation media. Furthermore, we compared lycopene production in modeling suggested media with other media designed based on compounds usually reported in the literature through fermentation scale up experiments.

## 2. Results

### 2.1. Media Designing for Bioprocess Improvement

#### FBA-Independent Screening of Medium Components

Up to date, no deep investigation for development of fermentation media for lycopene production from *Y. lipolytica* has been realized. In order to establish a reference medium for evaluation of the FBA efficiency, we first selected a set of 23 factors in the light of preliminary experiments and literature review for the designing of FBA-independent cultivation medium. Bioprocess improvement experiments were carried out using the engineered strain Po1f-1312E-1269IB. In a first step, we screened a set of chemicals without considering the flux balance analysis (FBA). The matrix of the Plackett-Burman design used in regard of this achievement is reported in Table 1. According to the Plackett-Burman principle, data transformation is required if the ratio max/min is greater than 10. For this reason, we used Log_10_ or the root square (Sqrt) for data transformation. Subsequently, the Pareto chart was used for analyzing the effects of different components and selecting those to be included in the model. There are two different *t* limits plotted on the graph based on the Bonferroni or family-wise corrected *t* and a standard *t* for individual effects tested. Effects above the Bonferroni Limit were added to the model as certainly significant factors. Effects above the *t*-value Limit were possibly significant and added if they make sense. Effects below the *t*-value limit were likely insignificant. Pareto charts of all experimental data are reported in Supplementary File 1.

The results showed that biomass production was significantly (effects above the Bonferroni Limit) improved by the effects of KNO_3_, MgSO_4_·7H_2_O, FeCl_3_·6H_2_O, FeSO_4_·7H_2_O, yeast extract, fructose, BaCl_2_·2H_2_O, NiCl_2_·6H_2_O, KH_2_PO_4_ and vitamin B_1_ in this decreasing order. The effect of MnCl_2_·4H_2_O, Na_2_MoO_4_·2H_2_O and KI were also positively significant (*t*-value < effects < Bonferroni Limit) while the positive effect of K_2_HPO_4_ was not significant. The set of factors including CuCl_2_·2H_2_O, NiSO_4_·6H_2_O, CoCl_2_·6H_2_O, H_3_BO_3_, ZnCl_2_, MnSO_4_·7H_2_0, NaCl, Glucose, (NH_4_)_2_SO_4_ were found as inhibitors of biomass production. The highest biomass concentration obtained was 16.14 g/L with max/min value of 25.22. The final equation in term of coded factors was: Sqrt (Biomass) = 2.24 − 0.38 × A − 0.18 × B − 0.043 × C + 0.29 × D + 0.15 × E − 0.13 × G − 0.095 × H + 0.072 × J + 0.089 × K + 0.28 × L + 0.082 × M + 0.29 × N + 0.37 × O − 0.095 × Q +0.22 × R + 0.25 × S − 0.20 × T + 0.14 × U + 0.020 × V + 0.12 × W + 0.19 × X.

For lycopene production, MgSO_4_·7H_2_O, FeCl_3_·6H_2_O, fructose, KNO_3_, NiCl_2_·6H_2_O, vitamin B1 and KH_2_PO_4_ had significant positive effects. The maximum lycopene concentration obtained in flasks was 9.18 mg/L. The final equation in terms of coded factors was: Sqrt (Lycopene)= 0.75 − 0.52 × A − 0.12 × B − 0.2 × C + 0.32 × D + 0.18 × E + 0.059 × G + 0.074 × H + 0.081 × J + 0.052 × K + 0.049 × L + 0.049 × M + 0.3 × N + 0.26 × O − 0.064 × P − 0.071 × Q + 0.28 × R − 0.15 × T + 0.11 × U − 0.17 × V + 0.14 × W + 0.072 × X.

The maximal lycopene content obtained from the 24 runs was 1.11 mg/g dry cell weight (DCW). Lycopene content was positively promoted by the effects of MgSO_4_·7H_2_O, NiCl_2_·6H_2_O, ZnCl_2_, MnSO_4_·7H_2_0, KI, MnCl_2_·4H_2_O, FeCl_3_·6H_2_O, KNO_3_, fructose, KH_2_PO_4_, vitamin B1, BaCl_2_·2H_2_O. Other components had negative effects.

The post-optimization experiments conducted to the optimized medium (PM medium) with the following composition (g/L): MgSO_4_·7H_2_O (14.74), NiCl_2_·6H_2_O (0.097), H_3_BO_3_ (0.099), ZnCl_2_ (0.15), MnSO_4_·7H_2_0 (0.4), KI (0.012), MnCl_2_·4H_2_O (0.2), Na_2_MoO_4_·2H_2_O (0.1), FeCl_3_·6H_2_O (2.87), KNO_3_ (0.05), Glucose (19.9), fructose (20), yeast extract (0.96), (NH_4_)_2_SO_4_ (0.51), KH_2_PO_4_ (10) and vitamin B1 (1.00E-02).

**Table 1:** Matrix of Plackett-Burman screening of medium components selected independently from the FBA.

Std	Run	A:CuCl_2_·2H_2_O	B:NiSO_4_·6H_2_O	C:CoCl_2_·6H_2_O	D:MgSO_4_·7H_2_O	E:NiCl_2_·6H_2_O	F:H_3_BO_3_	G:ZnCl_2_	H:MnSO_4_·7H_2_0	J:KI	K:MnCl_2_·4H_2_O	L:FeSO_4_·7H_2_O	M:Na_2_MoO_4_·2H_2_O	N:FeCl_3_·6H_2_O	O:KNO_3_	P:NaCl	Q:Glucose	R:Fructose	S:Yeast extract	T:(NH_4_)_2_SO_4_	U:KH_2_PO_4_	V:K_2_HPO_4_	W:Vitamine B1	X:BaCl_2_·2H_2_O	Biomass	Lycopene	Lycopene content
			g/L	g/L	g/L	g/L	g/L	g/L	g/L	g/L	g/L	g/L	g/L	g/L	g/L	g/L	g/L	g/L	g/L	g/L	g/L	g/L	g/L	g/L	g/L	mg/L	mg/g
**10**	1	0.03	1	0.4	1.5	0.1	0.01	0.01	0.02	0.01	0.2	2.5	0.1	3	0.2	0.01	20	0	10	10	0.01	0	0.01	1	14.26	1.76	0.12
**22**	2	0.3	3	0.4	1.5	0.1	0.01	0.15	0.4	0.01	0.01	2.5	0.1	0.02	0.01	1	10	20	0.5	0.5	0.01	0	0.01	1	1.52	0	0
**12**	3	0.3	3	0.02	1.5	0.1	0.01	0.15	0.02	0.01	0.01	0.1	0.1	3	0.2	1	20	0	10	0.5	10	3.5	0	0.01	3.38	0	0
**2**	4	0.03	3	0.4	15	0.1	0.1	0.01	0.4	0.01	0.2	2.5	0.01	0.02	0.2	1	10	0	10	0.5	10	0	0	0.01	11.06	1.82	0.16
**7**	5	0.03	3	0.02	1.5	0.05	0.01	0.15	0.4	0.1	0.2	2.5	0.01	3	0.01	1	20	0	0.5	10	10	0	0	1	2.36	0.6	0.26
**13**	6	0.03	3	0.4	1.5	0.05	0.1	0.01	0.4	0.01	0.01	0.1	0.01	3	0.2	1	20	20	0.5	10	0.01	3.5	0.01	0.01	2.66	0.37	0.14
**1**	7	0.3	3	0.4	15	0.1	0.01	0.15	0.02	0.1	0.2	0.1	0.01	3	0.2	0.01	10	20	0.5	10	0.01	0	0	0.01	3.46	1.17	0.34
**16**	8	0.3	3	0.02	1.5	0.1	0.1	0.01	0.02	0.1	0.01	2.5	0.01	0.02	0.01	0.01	20	20	10	10	10	0	0.01	0.01	2.38	0	0
**3**	9	0.03	1	0.4	15	0.1	0.1	0.15	0.02	0.1	0.01	2.5	0.1	0.02	0.01	1	20	0	0.5	10	0.01	3.5	0	0.01	2.74	0.04	0.02
**15**	10	0.3	1	0.02	15	0.1	0.01	0.01	0.4	0.01	0.2	0.1	0.01	0.02	0.01	1	20	20	10	10	0.01	3.5	0	1	2.82	0	0
**14**	11	0.03	1	0.4	15	0.05	0.01	0.15	0.02	0.1	0.01	0.1	0.01	0.02	0.2	1	20	20	10	0.5	10	0	0.01	1	12.84	3.82	0.3
**21**	12	0.3	3	0.02	15	0.05	0.1	0.15	0.02	0.01	0.2	2.5	0.01	0.02	0.2	0.01	20	0	0.5	0.5	0.01	3.5	0.01	1	3.76	0.04	0.01
**24**	13	0.03	1	0.02	1.5	0.05	0.01	0.01	0.02	0.01	0.01	0.1	0.01	0.02	0.01	0.01	10	0	0.5	0.5	0.01	0	0	0.01	0.64	0	0
**18**	14	0.3	1	0.4	15	0.05	0.01	0.15	0.4	0.01	0.01	2.5	0.01	3	0.01	0.01	10	0	10	10	10	3.5	0.01	0.01	3.52	0	0
**19**	15	0.03	3	0.02	15	0.1	0.01	0.01	0.4	0.1	0.01	0.1	0.1	0.02	0.2	0.01	10	0	0.5	10	10	3.5	0.01	1	7.36	2.82	0.38
**6**	16	0.3	1	0.02	1.5	0.05	0.1	0.15	0.4	0.1	0.2	0.1	0.1	0.02	0.2	1	10	0	10	10	0.01	0	0.01	0.01	1.38	0	0
**23**	17	0.3	3	0.4	15	0.05	0.1	0.01	0.4	0.1	0.01	0.1	0.1	3	0.01	0.01	20	0	10	0.5	0.01	0	0	1	2.4	0	0
**5**	18	0.03	1	0.02	1.5	0.1	0.1	0.15	0.4	0.1	0.01	2.5	0.01	3	0.2	0.01	10	20	10	0.5	0.01	3.5	0	1	16.14	5.76	0.36
**8**	19	0.3	1	0.4	1.5	0.05	0.01	0.01	0.4	0.1	0.2	2.5	0.1	0.02	0.2	0.01	20	20	0.5	0.5	10	3.5	0	0.01	4.44	0	0
**17**	20	0.03	3	0.4	1.5	0.05	0.1	0.15	0.02	0.01	0.2	0.1	0.1	0.02	0.01	0.01	10	20	10	10	10	3.5	0	1	2.76	0	0
**4**	21	0.03	1	0.02	15	0.1	0.1	0.15	0.4	0.01	0.2	0.1	0.1	3	0.01	0.01	20	20	0.5	0.5	10	0	0.01	0.01	8.3	9.18	1.11
**11**	22	0.3	1	0.02	15	0.05	0.1	0.01	0.02	0.01	0.01	2.5	0.1	3	0.2	1	10	20	0.5	10	10	0	0	1	11.26	2.17	0.19
**9**	23	0.03	3	0.02	15	0.05	0.01	0.01	0.02	0.1	0.2	2.5	0.1	3	0.01	1	10	20	10	0.5	0.01	3.5	0.01	0.01	15.16	3.27	0.22
**20**	24	0.3	1	0.4	1.5	0.1	0.1	0.01	0.02	0.1	0.2	0.1	0.01	3	0.01	1	10	0	0.5	0.5	10	3.5	0.01	1	4.36	0	0

### 2.2. Combination of Flux Balance Analysis and Plackett-Burman Design for Media Designing

#### 2.2.1. Flux Balance Analysis and Prediction of Media Components

In this study, the flux balance analysis (FBA) was implemented to determine metabolic flux distribution with the genome-scale metabolic network model of *Y. lipolytica* (yli v1.7) that was updated from model iYL619_PCP by correcting topological network and parameters of model (model yli v1.7 is available at http://www.echaoceshi.com/ECLOSB/CN/Default.aspx). In model yli v1.7, R0763 is the only reaction, in which farnesyl diphosphate (FPP) is produced; R2000 is the reaction of growth (biomass formation reaction). Besides, the maximum flux of R2000 (specific growth rate) is 0.0350 h^−1^ in the glucose minimal media. The model yli v1.7 and iYL619_PCP predicted the equal glucose minimal media. The composition of minimal media in the experiment is as follows: glucose (20 g/L), NH_4_SO_4_ (3 g/L), KH_2_PO_4_ (2 g/L), in which the maximum specific growth rate is 0.0352 h^−1^. This indicates that model yli v1.7 predicted the maximum specific growth rate more accurately (Supplementary File 2).

The fluxes of exchange reactions of the updated model were generated through FBA. Exchange reactions with negative fluxes indicated compounds that needed to be consumed when targeting a maximized production of FPP and the addition of those components into the culture medium was of crucial requirement. To predict the potential benefit of supplementing the minimal media with of amino acids or several cofactors in terms of FPP production based on FBA metabolic flux distributions, we relaxed the exchange reactions of model yli v1.7. The list of exchange reactions with negative fluxes and their fluxes when glucose, fructose, or both carbohydrates were selected as carbon sources is summarized in Supplementary File 3 (Table S1 of Supplementary File 3). The most prevailing class of exchange reactions included exchange reactions involving amino acids such as l-isoleucine, l-leucine, l-valine, l-asparagine, l-histidine, l-methionine, l-tryptophan, guanine, and l-lysine. The additional exchange reactions were those of O_2_, thiamine diphosphate, succinate, phosphate, ammonia, 4-aminobutanoate, ergosterol, ethanolamine, d-fructose, urea, d-glucose, hypoxanthine, and (R)-pantothenate. The number of negative exchange reactions and fluxes varied widely with the nature of the carbon source opted for. Putting aside the fact that FBA only gives one possible random solution instead of looking at all the possibilities (like Flux Variability Analysis would do, for example), it seems that the only “non-zero” values in this table are those corresponding to O_2_ exchange, d-fructose exchange and d-glucose exchange. All other values from −8.05e-35 to −1.46e-13 are most probably rounding errors of the LP solver, and so all those values could be considered as zeros. However, in order to predict the potential benefit of supplementing the minimal media with of above amino acids or several cofactors in terms of FPP production based on FBA metabolic flux distributions, we relaxed the exchange reactions of model yli v1.7. We first tested effects of 18 amino acids (only 18 amino acid can be utilized in model yli v1.7) on FPP production in a definite specific growth rate (90% of the maximum specific growth rate), and found that all the 18 amino acids facilitated FPP production in different degrees (Table 2). However, only eight amino acids (l-isoleucine, l-leucine, l-valine, l-asparagine, l-histidine, l-methionine, l-lysine, l-tryptophan) were found as amino acids that could be supplemented to the minimal medium owing to the guarantee of C/N ratio more than 10. Additionally, we also tested effects of 10 factors on FPP production in a definite specific growth rate (Table 3). We found that a certain number of factors had significant effect on FPP production. Furthermore, all 10 factors were chosen and supplemented to the minimal medium to verify their effects on FPP production (Supplementary File 3).

**Table 2:** Fluxes (mmol/g DCW/h) of 18 exchange reactions of amino acids and their respective effects on FPP production.

ID	Reaction Name	Biomass	FPP	Flux
R1202	l-isoleucine exchange	0.0315 ^#^	0.4342	−0.0042
R1203	l-leucine exchange	0.0315	0.4352	−0.0085
R1205	l-valine exchange	0.0315	0.4350	−0.0104
R1226	l-glutamate exchange	0.0315	0.4763	−2 ^$^
R1241	l-alanine exchange	0.0315	0.5871	−2
R1244	l-arginine exchange	0.0315	0.5159	−2
R1245	l-asparagine exchange	0.0315	0.4344	−0.0052
R1248	l-cysteine exchange	0.0315	0.5878	−2
R1268	l-histidine exchange	0.0315	0.4347	−0.0030
R1273	l-methionine exchange	0.0315	0.4352	−0.0072
R1275	l-lysine exchange	0.0315	0.4353	−0.0067
R1280	l-serine exchange	0.0315	0.6640	−2
R1283	l-threonine exchange	0.0315	0.7025	−2
R1285	l-tryptophan exchange	0.0315	0.4339	−0.0010
R1298	l-aspartate exchange	0.0315	0.6640	−2
R1300	l-phenylalanine exchange	0.0315	0.6215	−2
R1301	l-proline exchange	0.0315	0.5150	−2
R1303	l-tyrosine exchange	0.0315	0.5650	−1.6980

^#^ we set a definite specific growth rate of 0.0315 h^−1^ at the 90% of the maximum specific growth rate; ^$^ we respectively set the lower bounds of amino acids exchange reaction to −2 mmol/g DCW/h.

**Table 3:** Fluxes (mmol/g DCW/h) of 10 exchange reactions of factors used to test effects on FPP production.

ID	Reaction name	Biomass	FPP	Flux
R1207	Thiamine diphosphate exchange	0.0315 ^#^	0.4332	0.0000
R1239	4-aminobutanoate exchange	0.0315	0.4525	−1 ^$^
R1251	Ergosterol exchange	0.0315	0.4333	−0.0002
R1258	Ethanolamine exchange	0.0315	0.4392	−0.0769
R1267	Guanine exchange	0.0315	0.4345	−1
R1278	(R)Pantothenate exchange	0.0315	0.4332	0.0000
R1281	Sulfite exchange	0.0315	0.4332	0
R1287	Urea exchange	0.0315	0.4332	0
R1296	Hypoxanthine exchange	0.0315	0.4353	−0.0049
R1306	Pyridoxine exchange	0.0315	0.4332	0

^#^ we set a definite specific growth rate of 0.0315 h^−1^ at the 90% of the maximum specific growth rate; ^$^ we respectively set the lower bounds of factors exchange reaction to −1 mmol/g DCW/h.

#### 2.2.2. Plackett-Burman Design Screening of FBA-Predicted Components

Taking into account the results of the above simulations, we applied the Plackett-Burman design for experimental validation of FBA-predicted factors. The design matrix and responses obtained for biomass, lycopene and lipid production in Plackett-Burman design screening of FBA-predicted factors are correspondingly reported in Table 4. The results showed that biomass production ranged from 9.075 to 35.5 g/L (ratio max/min = 3.91) while the produced lycopene and lipid concentrations ranged from 1.77 to 41.23 mg/L (ratio max/min = 23.29) and 0.82 to 2.65 g/L (ratio max/min = 3.23), respectively. The highest lycopene and lipid contents were respectively 2.9 mg/g (ratio max/min = 29.48) and 12.01% (ratio max/min = 4.48). On media corresponding to run 7 and run 8, the engineered strain of *Y. lipolytica* produced consistent amount of lycopene (41.23 and 40.54 mg/L, respectively) with average biomass production of 14.23 and 16.53 g/L leading to lycopene content of 2.90 and 2.45 mg/g. Approximately equal quantities of lipids (1.55 and 1.53 g/L) were yielded on both media and corresponded to lipid contents of 10.90% and 9.23%.

The analysis of the effects of FBA-predicted factors on the biomass production showed that thiamine pyrophosphate, nicotinate, KH_2_PO_4_, ethanolamine, guanine, l-methionine, l-tryptophan, l-valine, l-asparagine, d-glucose, and d-fructose had positive effects on biomass production. Fructose was the most significant terms (effect above the Bonferroni Limit) followed by KH_2_PO_4_, l-valine, ethanolamine, nicotinate, l-asparagine, guanine, l-tryptophan, and glucose. The list of compounds composed of pyridoxine hydrochloride, succinate, 4-aminobutyric acid, Na_2_SO_3_, hypoxanthine, l-histidine, l-isoleucine, and l-leucine had negative effects on biomass production. The negative effects of 4-aminobutyric acid, l-histidine, and l-leucine were not significant. The effects of thiamine pyrophosphate and l-methionine were not significant for biomass production. The final equation of biomass production obtained after ANOVA analysis in terms of coded factors was:

Biomass = 1761.61 +1.00 × A − 1.16 × B + 1158.95 × C − 1.25 × D − 0.77 × E +2.70 × F − 1.70 × J + 1.57 × L − 1.18 × M + 1.51 × N − 0.71 × O + 0.63 × P + 1.41 × R + 1.75 × S + 1.52 × T − 1.85 × U − 0.66 × V + 1.14 × W + 3.28 × X.

Six factors were found to have significant positive effects on lycopene production. The most significant factors were l-tryptophan, l-isoleucine, ergosterol, and thiamine pyrophosphate (effect above the Bonferroni Limit) followed by ethanolamine and (NH_4_)_2_SO_4_ (effects above the *t*-value Limit). Although l-asparagine had a positive effect, this effect was not significant. All other compounds had an inhibitory effect on lycopene production. The final equation in terms of coded factors was:

Log_10_ (Lycopene) = 1.10 + 0.055 × A − 0.049 × B − 0.14 × D − 0.056 × E − 0.046 × F + 0.026 × G − 0.15 × J + 0.080 × K + 0.049 × L − 0.11 × M − 0.010 × N − 0.085 × P + 5.397e-004 × Q + 0.14 × R − 0.027 × S + 8.320e-003 × T + 0.095 × U − 0.14 × V − 0.053 × W − 0.077 × X.

l-isoleucine was found as the most significant factor having positive effect on lycopene content, followed by l-tryptophan, ergosterol, thiamine pyrophosphate, and l-histidine. Ethanolamine and (NH_4_)_2_SO_4_ had negligible positive effects on lycopene content. The large number of factors negatively influenced the lycopene content.

**Table 4:** Matrix of Plackett-Burman design of FBA predicted medium components.

Std	Run	A:Thiamine pyrophosphate	B:Pyridoxine, hydrocloride	C:Succinate	D:(R) Pantothenate	E:4-Aminobutyric acid	F:KH_2_PO_4_	G:(NH_4_)_2_SO_4_	H:Urea	J:Sulfite Na_2_SO_3_	K:Ergosterol	L:Ethanolamine	M:Hypoxanthine	N:Guanine	O:l-histidine	P:l-methionine	Q:l-lysine	R:l-tryptophan	S:l-valine	T:l-asparagine	U:l-isoleucine	V:l-leucine	W:d-glucose	X:d-fructose	Lycopene	Biomass	Lycopene content	Lipid	Lipid content
		g/L	g/L	g/L	g/L	g/L	g/L	g/L	g/L	g/L	g/L	g/L	g/L	g/L	g/L	g/L	g/L	g/L	g/L	g/L	g/L	g/L	g/L	g/L	mg/L	g/L	mg/g	g/L	%
12	1	0.006	0.006	1	1	5	6	2	0.5	0.1	0.01	0.05	5	5	5	5	5	1	5	1	10	10	20	20	9.99	13.35	0.75	1.27	9.51
16	2	0.006	0.006	1	1	5	12	0.5	0.5	2	0.01	0.3	1	1	1	1	5	5	5	10	10	1	60	20	34.51	26.73	1.29	2.41	9.02
4	3	0.0006	0.0006	1	5	5	12	2	2	0.1	0.1	0.05	5	5	1	1	5	5	1	1	10	1	60	20	27.87	19.48	1.43	2.2	11.3
6	4	0.006	0.0006	1	1	1	12	2	2	2	0.1	0.05	5	1	5	5	1	1	5	10	1	1	60	20	9.85	27.4	0.36	1.57	5.73
20	5	0.006	0.0006	5	1	5	12	0.5	0.5	2	0.1	0.05	1	5	1	5	1	1	1	1	10	10	60	60	6.69	30.88	0.22	1.6	5.18
22	6	0.006	0.006	5	1	5	6	2	2	0.1	0.01	0.3	5	1	1	5	1	5	1	1	1	1	60	60	18.47	31.45	0.59	1.02	3.24
8	7	0.006	0.0006	5	1	1	6	0.5	2	2	0.1	0.3	5	1	5	1	5	5	1	1	10	10	20	20	41.23	14.23	2.9	1.55	10.9
24	8	0.0006	0.0006	1	1	1	6	0.5	0.5	0.1	0.01	0.05	1	1	1	1	1	1	1	1	1	1	20	20	40.54	16.53	2.45	1.53	9.23
18	9	0.006	0.0006	5	5	1	6	2	2	0.1	0.01	0.3	1	5	1	1	1	1	5	10	10	10	60	20	19.95	29.05	0.69	1.63	5.61
13	10	0.0006	0.006	5	1	1	12	0.5	2	0.1	0.01	0.05	1	5	5	5	5	5	1	10	1	10	60	20	12.41	30.58	0.41	0.82	2.68
3	11	0.0006	0.0006	5	5	5	12	2	0.5	2	0.01	0.3	5	1	1	5	5	1	1	10	1	10	20	20	2.07	21.05	0.1	1.45	6.86
5	12	0.0006	0.0006	1	1	5	12	2	2	2	0.01	0.3	1	5	5	1	1	5	5	1	1	10	20	60	9.8	33.23	0.3	1.4	4.21
7	13	0.0006	0.006	1	1	1	6	2	2	2	0.1	0.3	1	5	1	5	5	1	1	10	10	1	20	60	23.58	23.65	1	1.62	6.85
2	14	0.0006	0.006	5	5	5	12	0.5	2	0.1	0.1	0.3	1	1	5	5	1	1	5	1	10	1	20	20	16.44	21.33	0.77	0.88	4.13
19	15	0.0006	0.006	1	5	5	6	0.5	2	2	0.01	0.05	5	1	5	1	1	1	1	10	10	10	60	60	1.77	9.08	0.2	1.09	12.01
9	16	0.0006	0.006	1	5	1	6	0.5	0.5	2	0.1	0.3	5	5	1	5	1	5	5	1	1	10	60	20	4.09	21.3	0.19	1.56	7.3
15	17	0.006	0.0006	1	5	5	6	0.5	2	0.1	0.1	0.05	1	1	1	5	5	5	5	10	1	10	20	60	12.36	29.13	0.42	1.44	4.93
14	18	0.0006	0.0006	5	5	1	6	2	0.5	2	0.01	0.05	1	1	5	5	5	5	5	1	10	1	60	60	11.14	24.75	0.45	1.56	6.3
10	19	0.0006	0.0006	5	1	5	6	0.5	0.5	0.1	0.1	0.3	5	5	5	1	5	1	5	10	1	1	60	60	16.32	35.5	0.46	1.89	5.32
1	20	0.006	0.006	5	5	5	6	2	0.5	2	0.1	0.05	1	5	5	1	1	5	1	10	1	1	20	20	24.84	20.73	1.2	2	9.65
21	21	0.006	0.006	1	5	1	12	2	0.5	0.1	0.1	0.3	1	1	5	1	5	1	1	1	1	10	60	60	9.99	28.65	0.35	2.65	9.23
17	22	0.0006	0.006	5	1	1	12	2	0.5	0.1	0.1	0.05	5	1	1	1	1	5	5	10	10	10	20	60	24.79	32.63	0.76	2.22	6.8
11	23	0.006	0.0006	1	5	1	12	0.5	0.5	0.1	0.01	0.3	5	5	5	5	1	5	1	10	10	1	20	60	22.7	33.83	0.67	1.19	3.52
23	24	0.006	0.006	5	5	1	12	0.5	2	2	0.01	0.05	5	5	1	1	5	1	5	1	1	1	20	60	3.07	27.73	0.11	1.62	5.83

The final equation for lycopene content in terms of coded factors was:

Log_10_ (Lycopene content) = −42.56 + 0.033 × A − 0.025 × B − 28.22 × C − 0.12 × D − 0.035 × E − 0.10 × F + 0.012 × G − 0.12 × J + 0.067 × K + 0.015 × L − 0.073 × M − 0.041 × N + 0.020 × O − 0.10 × P + 0.10 × R − 0.065 × S − 0.017 × T + 0.14 × U − 0.12 × V − 0.072 × W − 0.13 × X.

For lipid production, ergosterol, (NH_4_)_2_SO_4_, and l-lysine had significant positive effects. The effects of KH_2_PO_4_, glucose, and thiamine pyrophosphate were also moderately significantly positive. l-methionine, urea, l-histidine, nicotinate, and hypoxanthine significantly inhibited lipid production. The effects of other components were not significant. The final equation for lycopene content in terms of coded factors was: 

Lipid = −76.10 + 0.072 × A − 51.85 × C − 0.036 × E + 0.077 × F + 0.13 × G − 0.19 × H + 0.029 × J + 0.17 × K − 0.038 × M − 0.023 × N − 0.10 × O − 0.26 × P + 0.12 × Q + 0.024 × R + 0.030 × S + 0.021 × T − 0.034 × V + 0.077 × W.

Lipid content was positively affected by l-isoleucine, Na_2_SO_3_, l-lysine, hypoxanthine, ergosterol, succinate, pyridoxine hydrochloride, 4-aminobutyric acid, (NH_4_)_2_SO_4_, and l-leucine. l-histidine had no significant positive effect. The rest of predicted components strongly repressed lipid accumulation.

Based on the above observations and multiple post-optimization experiments, the optimization process gave MP medium constituted of 1 g/L asparagine, 6 g/L KH_2_PO_4_, 0.5 g/L (NH_4_)_2_SO_4_, 0.1 g/L ergosterol, 0.3 g/L ethanolamine, 5 g/L lysine, 5 g/L tryptophan, 10 g/L isoleucine, 20 g/L fructose, and 10 g/L glucose.

#### 2.2.3. Analysis of the Correlation between Lipid Content and Lycopene Content

The correlation analysis was carried out using the Pearson correlation analysis method with a two-tailed test of significance. The correlation analysis was applied to the data obtained from the Plackett-Burman design responses obtained with the FBA-predicted factors. We obtained a Pearson correlation of 0.49 with a significance of 0.01. The correlation between both variable was significant at the 0.05 level. Furthermore, we found commonness between both variables when positive factors were taken into account. Specifically, lycopene content and lipid content were both significantly stimulated by l-isoleucine, ergosterol and (NH_4_)_2_SO_4_. This suggested the positive correlation between lipid accumulation and lycopene biosynthesis. This relationship between both pathways may be influenced by the composition of the culture medium since we found diverse discrete distribution of the concentration of these compounds in the different culture media. In addition, high lipid content did not necessarily imply high lycopene content.

#### 2.2.4. Analysis of the Effect of FBA Predicted Factors on the Expression of Genes of the Terpenoids Backbone

Following the first Plackett-Burman design (PBD), we conducted another PBD (Supplementary File 4) in order to adjust the culture medium components to optimized conditions and determine the effect of above selected positive factors on the terpenoids backbone genes through the determination of their expression levels. The PBD design responses demonstrated that biomass production was significantly improved by the effects of lysine, fructose, l-tryptophan, (NH_4_)_2_SO_4_, KH_2_PO_4_, thiamine, and l-isoleucine in this decreasing order. The rest of the components including glucose, l-asparagine, and ethanolamine had moderate negative effects on biomass production. The highest biomass production of 33.58 g/L was obtained with run 10. The elimination of factors with negative effects in the first PBD driven on the FBA predicted components did not significantly affect biomass production, showing that their elimination was not due to errors. Similarly, KH_2_PO_4_, fructose, ergosterol, (NH_4_)_2_SO_4_, and l-tryptophan promoted lycopene accumulation. A lycopene production of 4.35 mg/g DCW (76.62 mg/L) was achieved under the coordinated effect of these compounds in shake flasks. Only KH_2_PO_4_ had positive effect on lycopene content.

This second tour PBD suggested that the selected components were further optimizable for biomass and lycopene concentration but did not really impact on lycopene content. Furthermore, some of components with positive effects in the first round of screening were found inhibiting lycopene production. This could be explained by the fact that the scale of choice of these components was reduced.

In order to get insights into the effect of selected factors on genes of the terpenoids backbone, we realized quantitative RT-PCR experiments to explore expression levels of these genes. No significant effect was found for *YALI0B16126g* which is annotated as protein farnesyltransferase/geranylgeranyltransferase type-1 subunit alpha. On the contrary, significant effects were recorded for *YALI0C18755g*, the gene encoding for (2E,6E)-farnesyl pyrophosphate (FPP) specific hexaprenyl-diphosphate synthase. This gene was significantly stimulated by the effects of l-tryptophan, l-lysine, l-isoleucine, l-asparagine, KH_2_PO_4_, and fructose. The high number of positive factorial effects corroborated with the fact that FPP was set as the target metabolite in the FBA simulation. Only ergosterol, thiamine, and glucose had inhibitory effects on this gene. In addition, the ditrans, polycis-polyprenyl diphosphate synthase (also specific to (2E,6E)-farnesyl diphosphate) gene *YALI0C18799g* was activated by l-tryptophan, ergosterol, l-asparagine, and to some extent KH_2_PO_4_ while other components displayed negative effects. *YALI0D17556g*, another gene responsible for (2E,6E)-FPP specific ditrans, polycis-polyprenyl diphosphate synthase, was significantly stimulated by ethanolamine but repressed by l-tryptophan. The expression of the gene in charge of protein farnesyltransferase subunit beta, *YALI0D14762g*, was similarly triggered by l-lysine, l-asparagine, fructose, KH2PO4, and l-tryptophan and to a minor extent by glucose. Only l-tryptophan had a positive effect on *YALI0D17050g* (geranylgeranyl diphosphate synthase). The rate-limiting gene of the mevalonate pathway governing the expression of hydroxymethylglutaryl-CoA reductase (HMGCR), *YALI0E04807g*, was significantly activated by factors including l-lysine, l-asparagine, l-tryptophan, KH_2_PO_4_, and fructose but significantly inhibited by ergosterol and thiamine. The activation of this gene was necessary for converting HMG-CoA into mevalonic acid and subsequent metabolites of the terpenoids biosynthesis backbone. *YALI0E05753g* ((2E,6E)-farnesyl diphosphate synthase) was significantly stimulated by l-tryptophan, KH_2_PO_4_ and to a minor extent by L-asparagine. However, the lycopene biosynthesis genes introduced into the engineered strains, although highly expressed, were activated by numerous positive factors. However, the number of positive effects was not as significant as expected. This could be explained by the fact that the FBA was targeted towards FPP synthesis. Specifically, the expression of the *crtE*, *crtB*, and *crtI* genes were all promoted by ethanolamine (significant effect) and l-lysine, l-isoleucine, glucose, (NH_4_)_2_SO_4_, ergosterol, and glucose to a trivial degree.

**Figure 1:**
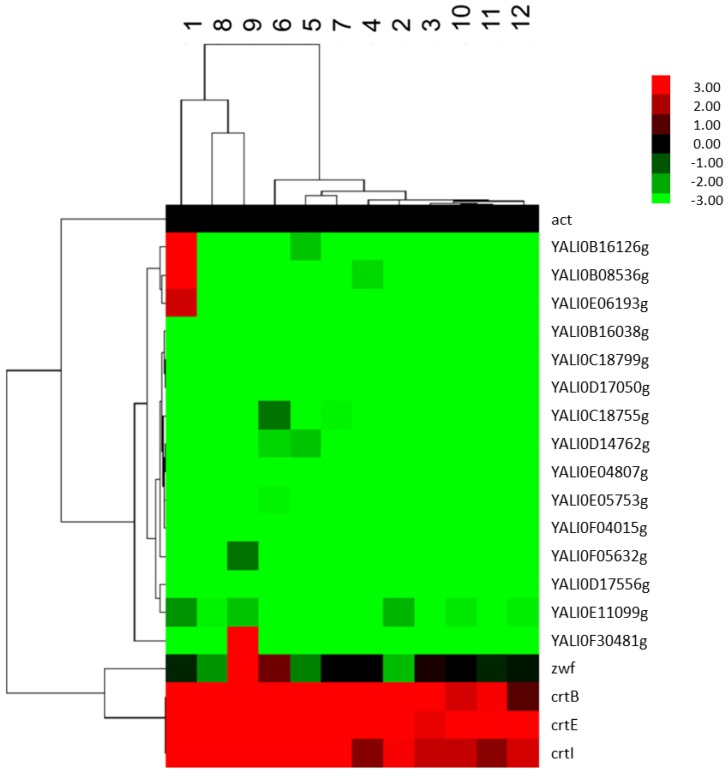
Heat map showing the expression levels of *crtE, crtB, crtI, zwf*, and terpenoids backbone genes in Po1f-1312E + 1269IB with respect to that of the internal control ACT1 on different culture media designed based on FBA predictions. The data represent the average of three independently grown cultures.

Overall, the results showed that FBA-predicted and PBD screened factors differentially affected genes of the terpenoids backbone, *zwf* and *crt* genes. Figure 1 is the heat map of the hierarchical clustering of genes relative expression levels obtained from the PBD experiments. We found that genes of the terpenoids backbone (Supplementary Figure S1) were downregulated after seven days of cultivation compared to the housekeeping gene *ACT1* while all heterologous genes were upregulated. The upregulation of heterologous genes implied that the carbon metabolism was efficiently directed to FPP biosynthesis and subsequently permitted lycopene production. Nevertheless, different amounts of biomass and lycopene yielded on different runs of culture media could be explained by divergences in expression patterns as depicted in Figure 1.

Indeed, though some heterologous genes were highly expressed in certain media, the level of produced lycopene did not match with expression levels. We found that some media components were inhibiting genes of the terpenoids backbone at different levels but the inhibition did not necessarily imply low production of lycopene. Each gene of the terpenoids backbone was activated by specific selected factors whose coordinated actions allowed high lycopene production.

### 2.3. Fed-Batch Fermentation of Y. lipolytica for Lycopene Production on Designed Media

**Figure 2:**
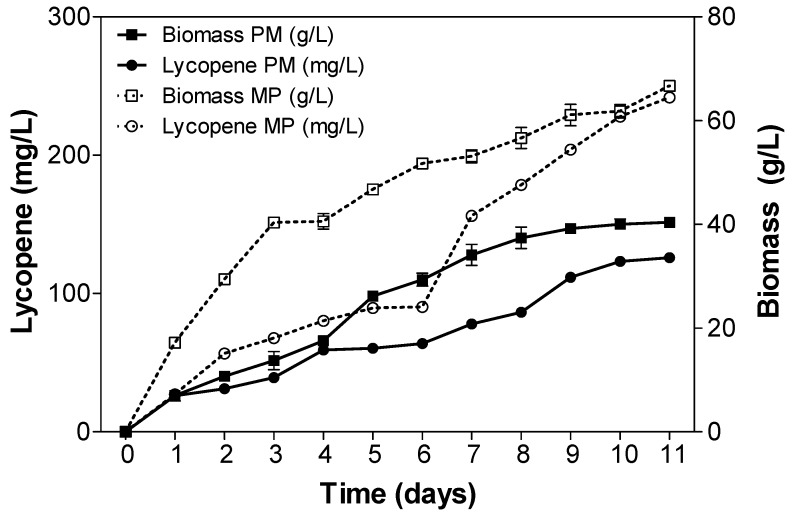
Fed-batch cultivation of Po1f-1312E + 1269IB for lycopene and biomass production in a 1.5 L fermenter at 30 °C and DO = 50% using the literature-based designed medium (PM) and the FBA-assisted (combination FBA + Plackett-Burman) designed medium (MP). Error bars embody standard deviation among the average of two distinct reiterated experiments. The pH was initially controlled at 5.5, then at 3 from the 48th hour of fermentation. The aeration rate was fixed at 1 vvm (volume air per volume per minute) and the agitation rate was set to automatically respond to the dissolved oxygen (DO) concentration so as to maintain it at 50%. The fed-batch started at 24 h with a feeding solution containing 200 g/L glucose and 200 g/L of fructose. The feeding was executed every 24 h with 50 mL of the feeding solution.

Designed media were initially used for lycopene production in a 1.5 L fermenter with a working volume of 1 L in batch. The fed-batch operation started at 24 h and consisted in the supplementation of a feeding solution containing 200 g/L glucose and 200 g/L of fructose. The feeding (50 mL) was executed every 24 h. The pH was initially controlled at 5.5 and then controlled at 3 after 48 h when the color began to appear. The results of biomass and lycopene production on different media over the fermentation course are depicted in Figure 2.

After 10 days of fermentation, we found a maximum biomass production of 40.2 g/L with a lycopene concentration of 126 mg/L from the medium designed without taking into account the FBA predictions (PM) (Figure 2a). Lycopene production increased progressively to reach a maximum value of 126 mg/L. In the medium obtained by combination of FBA and Plackett-Burman design (MP), a biomass of 61.08 g/L and a lycopene concentration of 242 mg/L were obtained after 10 days of fermentation (Figure 2b). Lycopene production in the designed using both methods (MP) was about two-fold that obtained in the PM medium. The maximum lycopene content of 4.02 mg/g DCW was obtained on this medium.

## 3. Discussion

Relative to other yeasts, *Y. lipolytica*, as an oleaginous yeast, is able to accumulate significant quantities of lipid bodies under certain growth conditions. This property provides a reliable storehouse for carotenoids biosynthesis and, consequently, the heterologous production of carotenoids using *Y. lipolytica* may constitute a promising asset for large-scale operations. The heterologous synthesis of lycopene in *Y. lipolytica* requires the introduction of the biosynthetic genes (*crtE*, *crtB* and *crtI*) even if the expression of *crtE* is not necessary in various heterologous hosts including *Y. lipolytica*, and bioengineering approaches for integrating these genes in *Y. lipolytica* have been described previously [24]. *CrtE* is a geranylgeranyl diphosphate synthase that catalyzes the conversion of FPP and IPP to GGPP and *CrtB* is a phytoene synthase that can condense two molecules of the C_20_ precursor, GGPP, to yield the first C_40_ hydrocarbon, the phytoene. This is followed by a series of sequential desaturations of phytoene to produce ζ-carotene, neurosporene, and, finally, lycopene [25]. This process is catalyzed by the phytoene desaturase (*CrtI*). The most advanced lycopene production using *Y. lipolytica* is the recent publication of Matthäus, Ketelhot, Gatter, and Barth [13] in which the codon-optimized genes *crtB* and *crtI* of *Pantoea ananatis* were expressed in *Y. lipolytica* under the control of the TEF1 promoter of *Y. lipolytica*. Moreover, these authors undertook the modifications of native metabolic pathways in *Y. lipolytica* via the overexpression of bottleneck genes in the isoprenoid pathway (GGS1 and HMG1) and the knock-out of *POX1* to *POX6* as well as *GUT2* genes and successfully enhanced lycopene production (16 mg/g DCW). In a previous study, taking into account the fact that the use of expression vectors pINA1269 and pINA1312, carotenoid genes *crtE*, *crtB*, and *crtI* can be successively integrated into the yeast chromosome with improved strain stability, we synchronically introduced combinations of these plasmids carrying lycopene biosynthesis genes and spawned lycopene producing strains. Further characterization of these engineered strains showed discrepancies in terms of lycopene and biomass production among engineered strains and suggested the impact of engineering tactics on lycopene biosynthesis in engineered strains.

In the present study, we aimed to establish an effective fermentation platform for lycopene production from the most robust engineered strain (Po1f-1312E + 1269IB) using statistical and GSMN modeling tools for screening chemical compounds and designing culture media. Efficient media were employed for fed-batch cultivation. The improvement of cultivation conditions (media and scale-up) led to lycopene production of 242 mg/L of lycopene using the medium designed based on the variables screened from the FBA-predicted factors. This concentration of lycopene was approximately the double of that obtained in the medium designed based on the literature. The above results demonstrated the usefulness of FBA and modelling tools for improved production of lycopene in consistency with previous investigations showing that FBA simulations can be efficiently applied for experimental validation and improvement of the production of objectives production or biological processes [26,27,28]. The lycopene content obtained in shake flasks was three-fold that achieved by Matthäus, Ketelhot, Gatter, and Barth [13] but the lycopene content in fed-batch cultivation mode was lower compared to their results. This could be explained by the fact that these authors carried out supplementary genetic modifications in their experiments or because of the source of lycopene biosynthesis genes. As a matter of fact, the lycopene production obtained from the present study was higher than the concentration these authors obtained with their basic strain (H222 Δ BI). In view of the above, we believe that the FBA-driven designed medium will contribute largely in the improvement of lycopene production. This could be applicable to a wide range of engineered strains of *Y. lipolytica* and other yeast species for lycopene and other terpenoids molecules. Additionally, our study gave insights into the comprehension of the effects of some external factors on genes of the terpenoids pathway. Correct monitoring of these factors for media designing, coupled with adequate metabolic engineering tactics, will contribute to the fundamental research and the efficient production of lycopene even at the industrial level.

## 4. Material and Methods

### 4.1. Description of the Engineered Lycopene Producing Y. Lipolytica Strain

Prior to cultivation improvement experiments, we first proceeded to the bioengineering of *Y. lipolytica* in order to obtain a dependable lycopene producing strain. The bioengineering work has been published in a local Chinese journal. The strain designated as Po1f-1312E + 1269IB, representing the recombinant strain obtained from the introduction of plasmid pINA1312 (carrying gene *crtE*) and pINA1269 (carrying genes *crtI* and *crtB*) into the host strain *Y. lipolytica* Po1f, was used in this study given that it displayed noteworthy growth and lycopene production behavior.

### 4.2. Modeling Platform

Flux balance analysis (FBA) is the paramount method for estimating flux distributions and assumes that organisms utilize substrates adequately to maximize a fixed objective of model (biomass or a particular reaction of production). The FBA method can be used to reproduce environmental conditions necessary for achieving the maximization of the objective function of the model and is an integrated element of the COBRA 2.0 Toolbox in Matlab. The FBA was carried out using Matlab R2011a (Mathworks, Natick, MA, USA) and the COBRA 2.0 toolbox. We used an *in silico Y. lipolytica* terpenoids biosynthesis genome-scale metabolic model (GSM) yli v1.7 updated from our previous publication [29]. The updated GSM yli v1.7 was composed of 718 genes, 1293 reactions, and 986 metabolites. The solvers used to optimize problems included “glpk” (version 4.35) and Tomlab/CPLEX (version 7.9). All the calculations were implemented on a personal computer with 3.40 GHz Intel (R) Core (TM) i7-2600k and 16.0 GB RAM. The implementation consisted in the computation of flux distributions with farnesyl pyrophosphate (FPP), a key terpenoids biosynthesis precursor, as the objective metabolite. Medium components were predicted by screening exchange reactions with negative fluxes.

### 4.3. Plackett-Burman Design and Shake Flasks Cultivation

To identify the vital factors that affect biomass and lycopene production in the engineered strain and make breakthrough improvements, a saturated screening design based on the Plackett-Burman structures incorporated into Design expert software (version 8) was adopted. The total number of trials to be implemented according to Plackett-Burman was (*n*+1) runs, wherein *n* represents the number of modules to be screened. Each component was assigned two levels of concentrations (high concentration and low concentration). Firstly, a set of 23 factors selected in light of the preliminary experiments and literature review (data not shown), were combined in 24 trials for the designing of FBA-independent cultivation media. Following this, the FBA-driven predicted factors (*n* = 23) were used for conducting a Plackett-Burman design of 24 runs. Numerous post-optimization experiments were equally carried out on the basis of Plackett-Burman design.

The cultivation conditions were as follows. Primarily, the metabolically engineered *Y. lipolytica* harboring carotenogenic plasmids were inoculated into 50 mL of S2 medium for 24 h at 30 °C with a shaking speed of 200 rpm. These pre-cultures were then transferred to a 250 mL shake flask containing 50 mL of each designed medium and grown for another seven days under the same fermentation temperature and agitation speed.

### 4.4. Bioreactor Scale-up

The optimized designed media were directly used for aerobic fermentation of the transgenic *Y. lipolytica* strain Po1f-1312E + 1269IB for lycopene production. The fermentation process was run in fed-batch mode in a 1.5 L stirred-tank fermenter (Baoxing Co., Shanghai, China) with a working volume of 1 L at 30 °C. The aeration rate was fixed at 1 vvm (volume air per volume per minute) and the agitation rate was set to automatically respond to the dissolved oxygen (DO) concentration so as to maintain it at 50%. Sterile air filters with 0.2 µm pores were used for air transfer into bioreactor. The DO concentration in the culture broth was measured using a pO_2_ electrode. Silicone was periodically added as an antifoam agent. The fed-batch operation started at 24 h and consisted in the supplementation of a feeding solution containing 200 g/L glucose and 200 g/L of fructose. The feeding (50 mL) was executed every 24 h. The pH was initially controlled at 5.5 and then controlled at 3 after 48 h following the appearance of color.

### 4.5. Determination of Glucose Concentration, Dry Cell Weight, and Lipids

After cultivation, 5 mL of cell broth was harvested, centrifuged at 12,000 rpm for 5 min, washed twice with distilled water and dried at 105 °C for 48 h. The dry cell weight (DCW) was determined by gravimetrically measuring the weight of the dry cell pellets. Glucose measurement was monitored by a spectrophotometric procedure by means of a Glucose Reagent Kit (Kexin Biotech Co., Ltd., Shanghai, China) following the vendor’s guidelines. Lipid extraction protocol was as described in our previous publication [30].

### 4.6. Lycopene Extraction and HPLC Analysis

Following the cultivation end time, precise volumes of homogenous culture broth were harvested, centrifuged at 12,000 rpm for 10 min at 4 °C and washed twice with deionized water. The supernatant was discarded and the pellet was used for lycopene extraction. Prior to lycopene extraction, pellets were dissolved in 500 μL of dimethyl sulfoxide (DMSO), pre-warmed at 55 °C for 30 min, shook strongly for 30 s, and then maintained for an extra 30 min without shaking. After that, 500 μL of acetone was added to the DMSO extract and homogenized by vortexing for 30 s, followed by incubation at 55 °C for 15 min with intermittent shaking. The mixture was subsequently centrifuged at 12,000 rpm for 10 min, and the colored mixed supernatants were transferred into a new tube. The extraction step was repeated until all visible pigments in the residual cell pellet and supernatant were extracted. The obtained colored supernatants were pooled together and used for lycopene quantification via high-performance liquid chromatography (HPLC) analysis. All operations occurred on ice under dim light to prevent photodegradation, isomerizations, and structural changes of the carotenoids.

The lycopene concentration of each extract was quantified by HPLC analysis which was performed on a Shimadzu LC-20A HPLC instrument equipped with a LC solution software program, LC-20AD pump, and a detector with a UV-Vis lamp. Specifically, it was carried out by injecting 10 μL of the crude carotenoid extract into a Zorbax SB-Aq C18-reverse-phase column (4.6 mm × 250 mm, 5 μm; Agilent Technologies, Palo Alto, CA, USA). The extract was eluted under isocratic condition with a mobile phase containing 80% acetonitrile, 15% methanol and 5% isopropanol at a flow rate of 1 mL/min at 30 °C. Quantitative analysis was accomplished using a calibration curve obtained with a lycopene standard and final lycopene concentrations were obtained by normalization to the total initial volumes used during extraction.

### 4.7. Quantitative Reverse Transcription-PCR (qRT-PCR) Studies

The cells were pelleted and total RNA was extracted as previously described [31]. To keep comparable content of starting materials, the total RNA was prudently determined with a DU 800 spectrophotometer (Beckman-Coulter, Fullerton, CA, USA). The chromosomal DNA was degraded by treatment with DNase I according to the manufacturer’s instructions. Next, 1000 ng of RNA from each sample was used for the reverse transcription with ReverTra Ace qPCR RT Master Mix instrument (Toyobo Co., Ltd., Osaka, Japan). Subsequently, 1 μL sample from each reaction mixture was used to perform quantitative PCRs (qPCRs) (in triplicate) with SYBR Green Realtime PCR Master Mix (Toyobo) using specific primers, with a non-template control. The primers used are presented in Supplementary File 5. The reactions were performed in a CFX Fast real-time PCR system (Bio-Rad Laboratories, Inc., Hercules, CA, USA).

To prevent the variation of starting materials amounts, *ACT1* was utilized as a housekeeping gene. The results were expressed as fractions of gene expression between the target gene and the housekeeping gene, *ACT1* [32].

## 5. Conclusions

The FBA and Plackett–Burman screening investigational design empowered the bringing about of significant variables for lycopene production from *Y. lipolytica*. The findings of the present study could have implications for improved production of lycopene using *Y. lipolytica* as a fermentation platform and promote terpenoids production at the industrial scale. Supplementary material in relation to Pareto charts of Plackett-Burman design and the Plackett-Burman design regarding terpenoids backbone genes expression levels is available on the journal website.

## Supplementary Files

Supplementary File 1
