# Supplementary material for: Flux Balance Analysis Inspired Bioprocess Upgrading for Lycopene Production by a Metabolically Engineered Strain of Yarrowia lipolytica
Source: Metabolites. 2015 Dec 21;5(4):794–813. doi: 10.3390/metabo5040794 (PMC4693195; doi:10.3390/metabo5040794)
Supplement: Supplementary File 1 [file metabolites-05-00794-s001.zip › metabolites-99322-supplementary-final/Supplementary File 1.pdf]

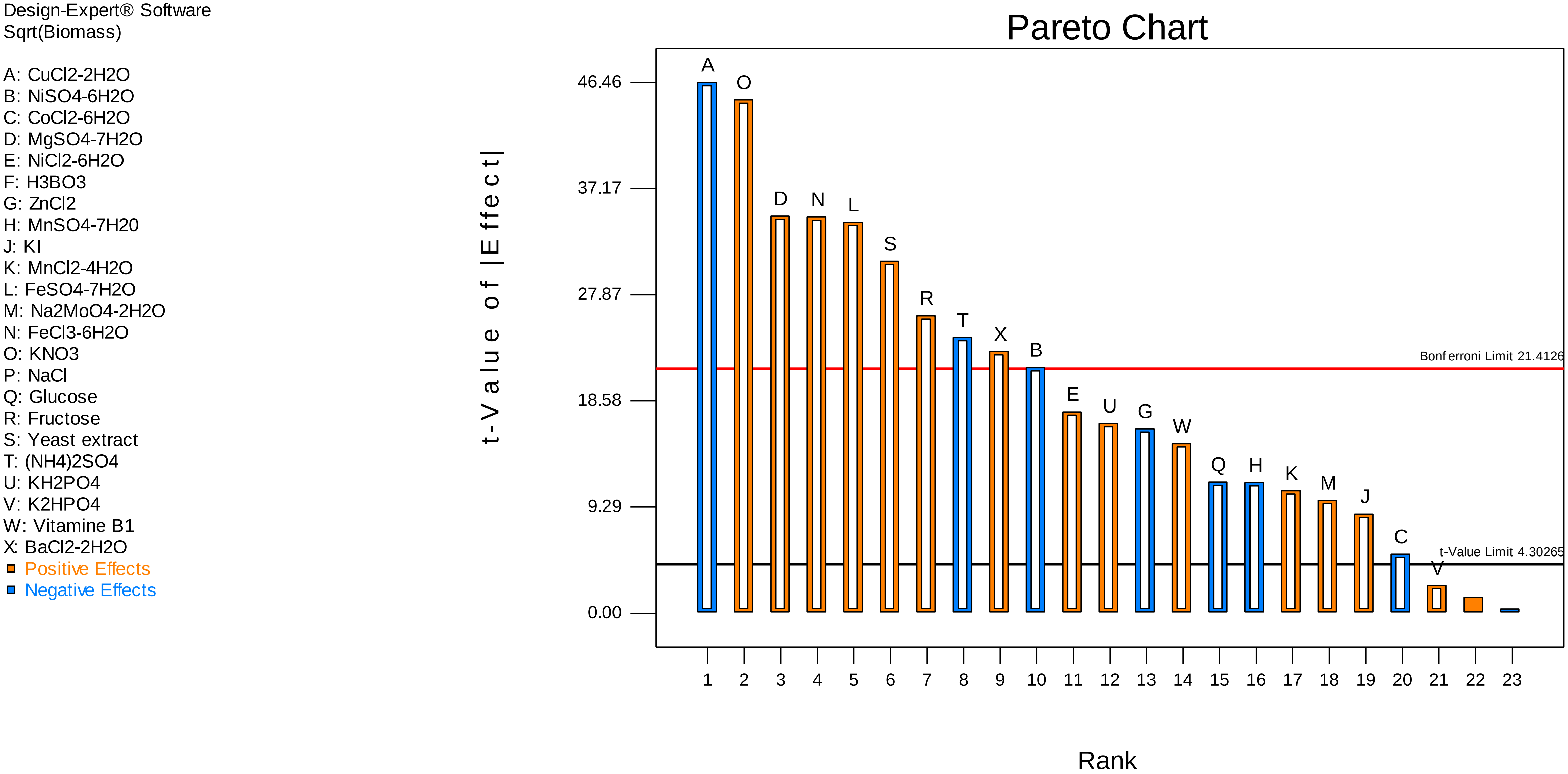

Fig S1: Pareto chart of the effects of chemical compounds selected according literature and preliminary experiments on biomass production by the engineered strain Po1f-1312E+1269IB

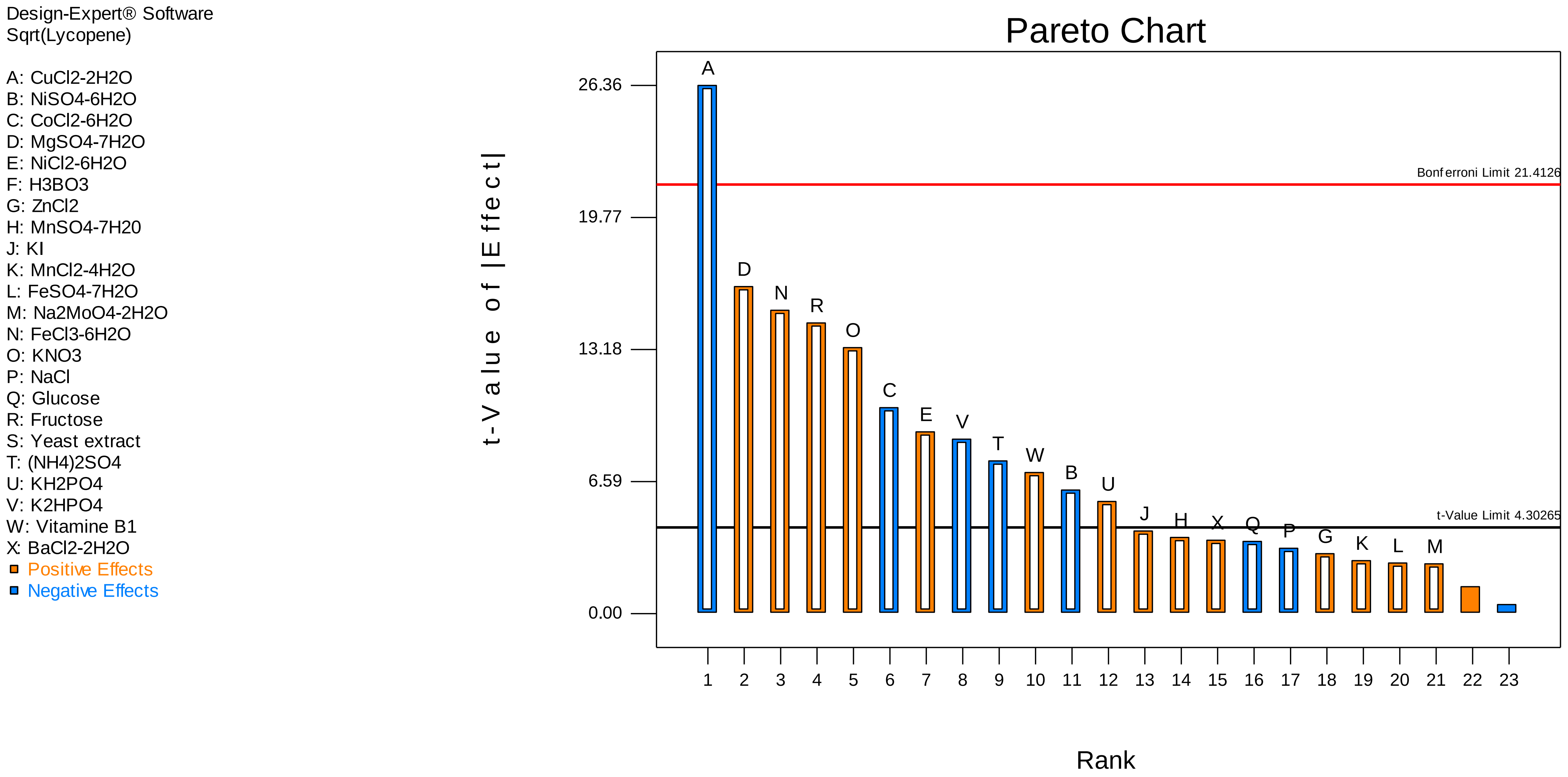

Fig S2: Pareto chart of the effects of chemical compounds selected according literature and preliminary experiments on lycopene production by the engineered strain Po1f-1312E+1269IB

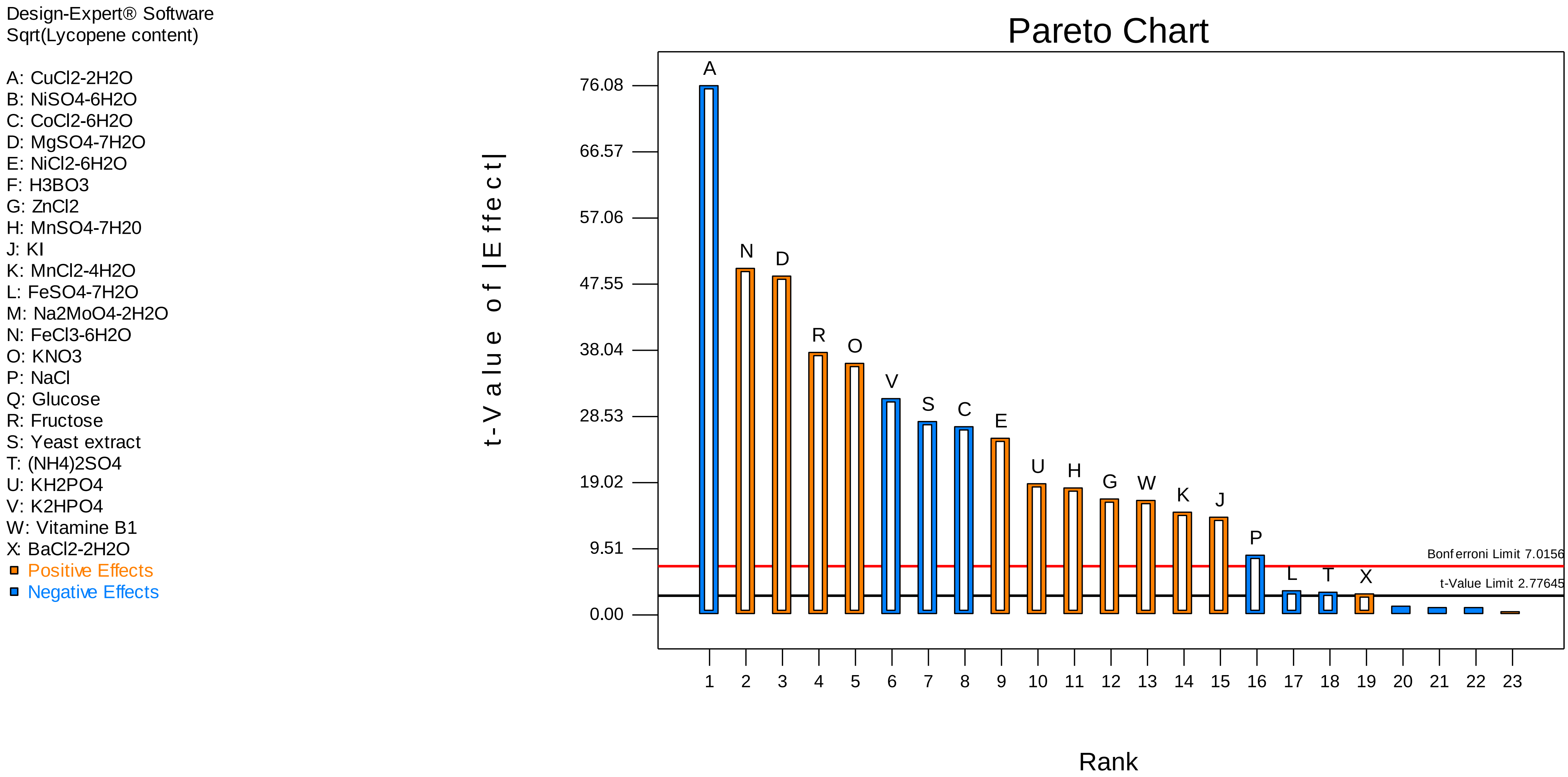

Fig S3: Pareto chart of the effects of chemical compounds selected according literature and preliminary experiments on lycopene content of the engineered strain Po1f-1312E+1269IB

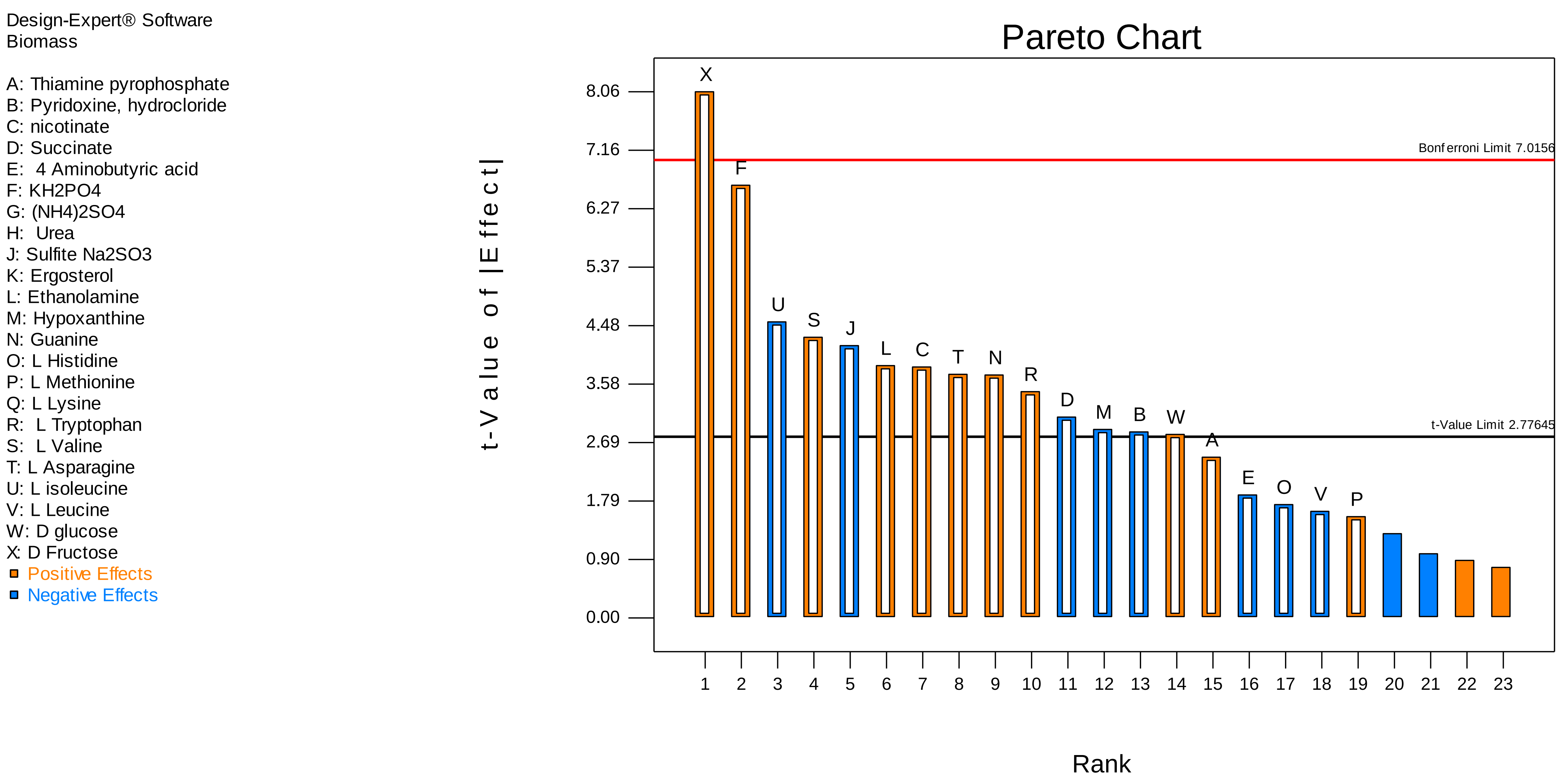

Fig S4: Pareto chart of the effects of chemical compounds predicted by flux balance analysis (FBA) on biomass production by the engineered strain Po1f-1312E+1269IB

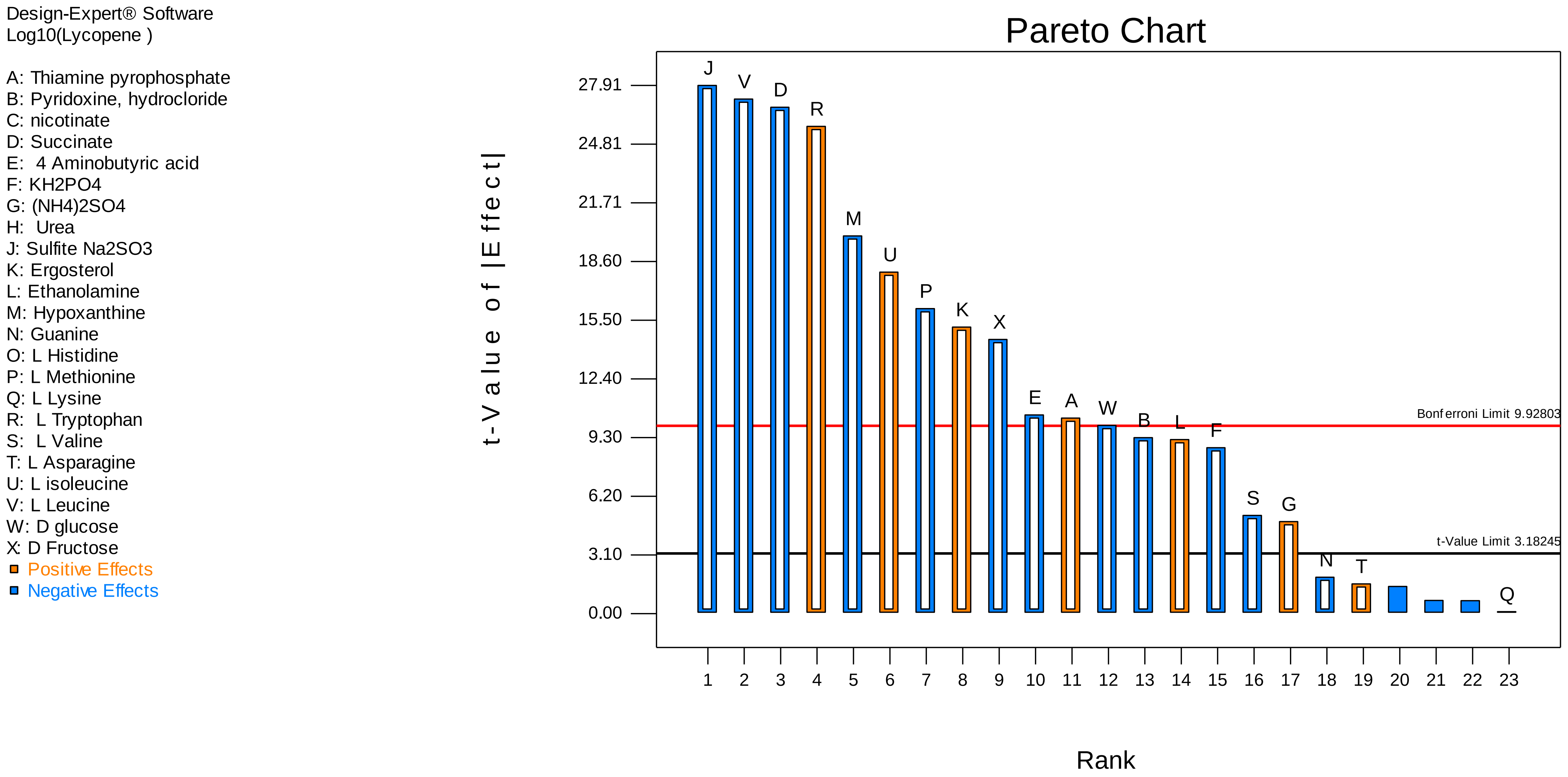

Fig S5: Pareto chart of the effects of chemical compounds predicted by flux balance analysis (FBA) on lycopene production by the engineered strain Po1f-1312E+1269IB

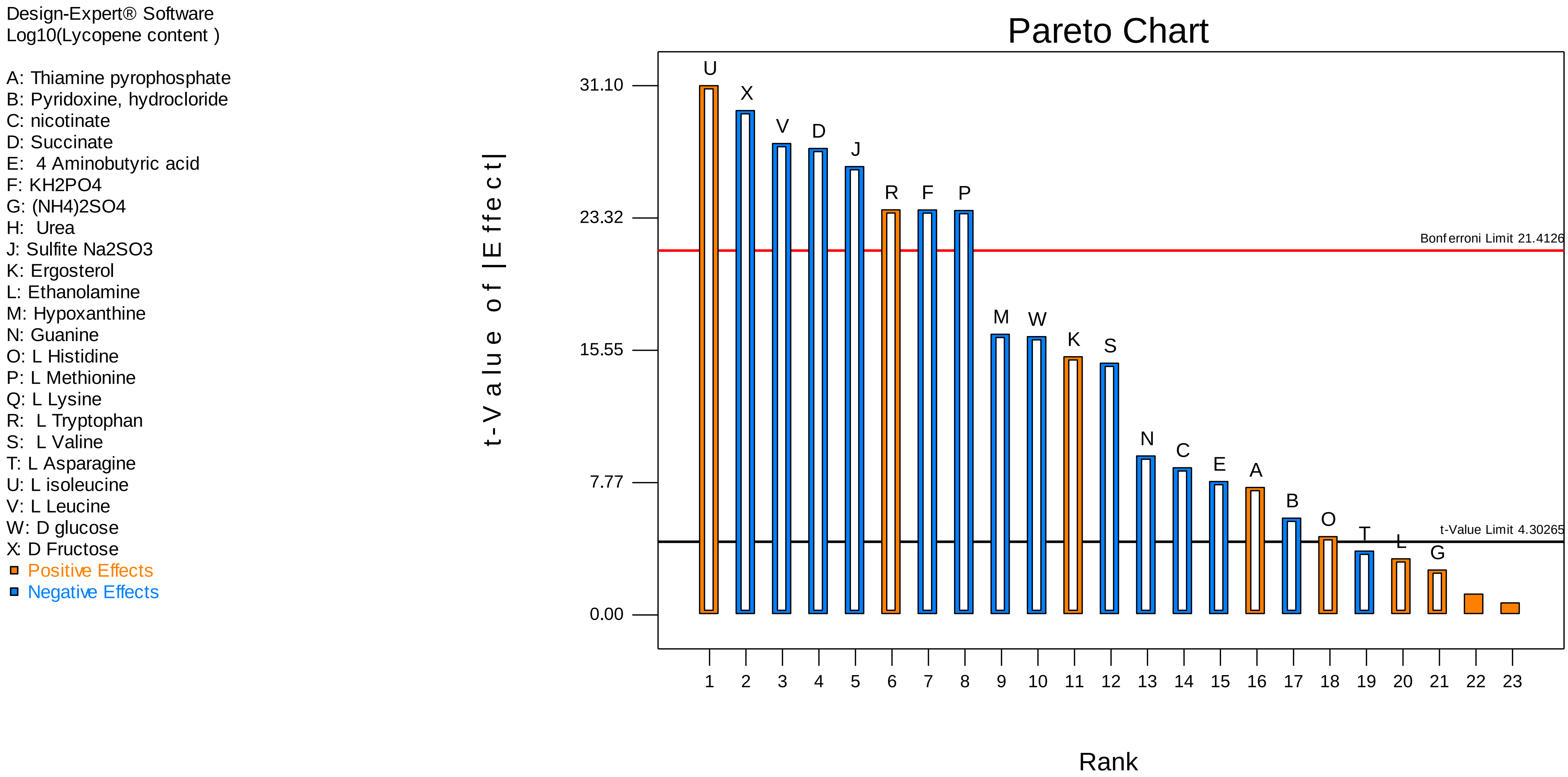

Fig S6: Pareto chart of the effects of chemical compounds predicted by flux balance analysis (FBA) on lycopene content of the engineered strain Po1f-1312E+1269IB

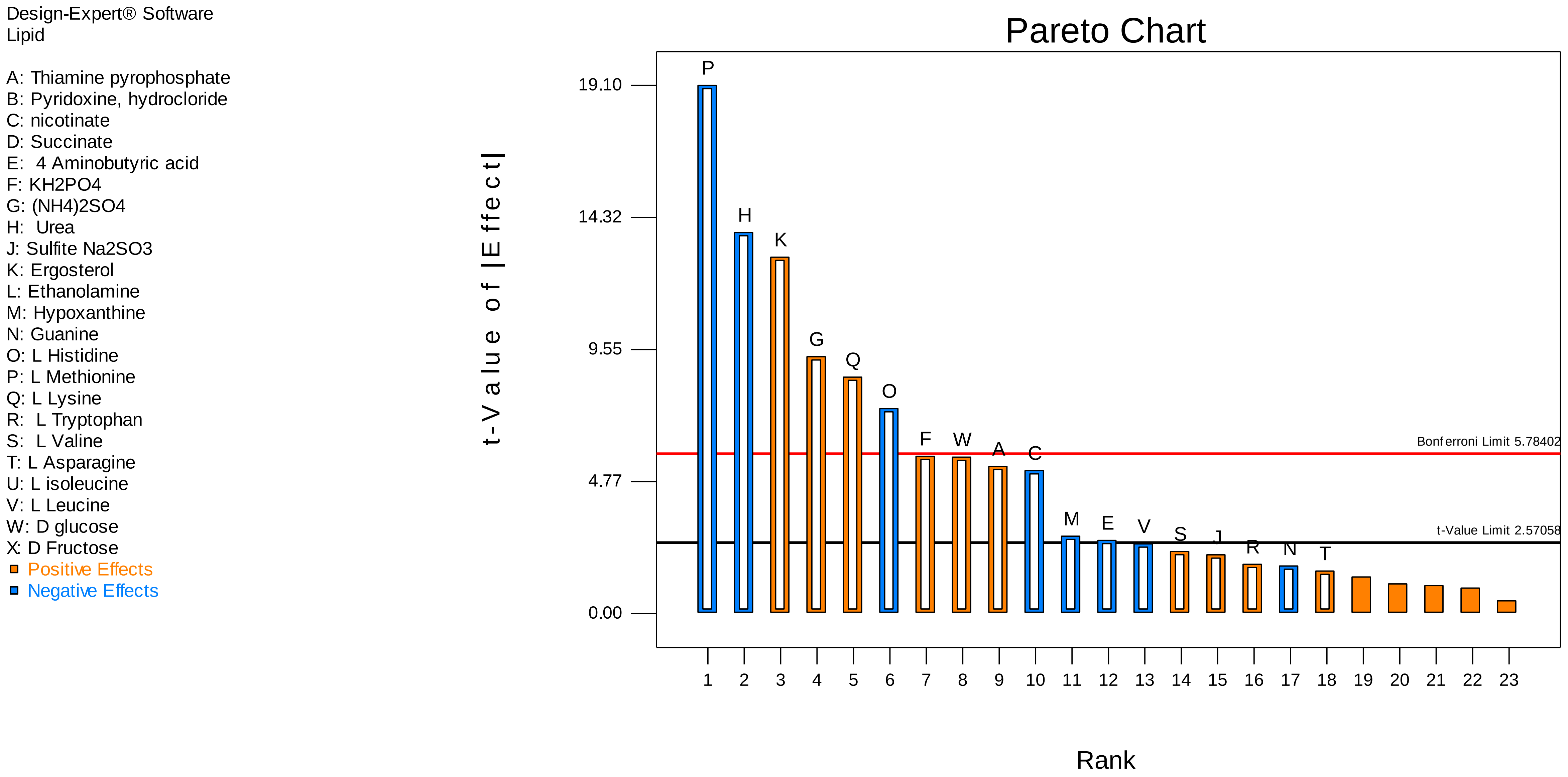

Fig S7: Pareto chart of the effects of chemical compounds predicted by flux balance analysis (FBA) on lipid production by the engineered strain Po1f-1312E+1269IB

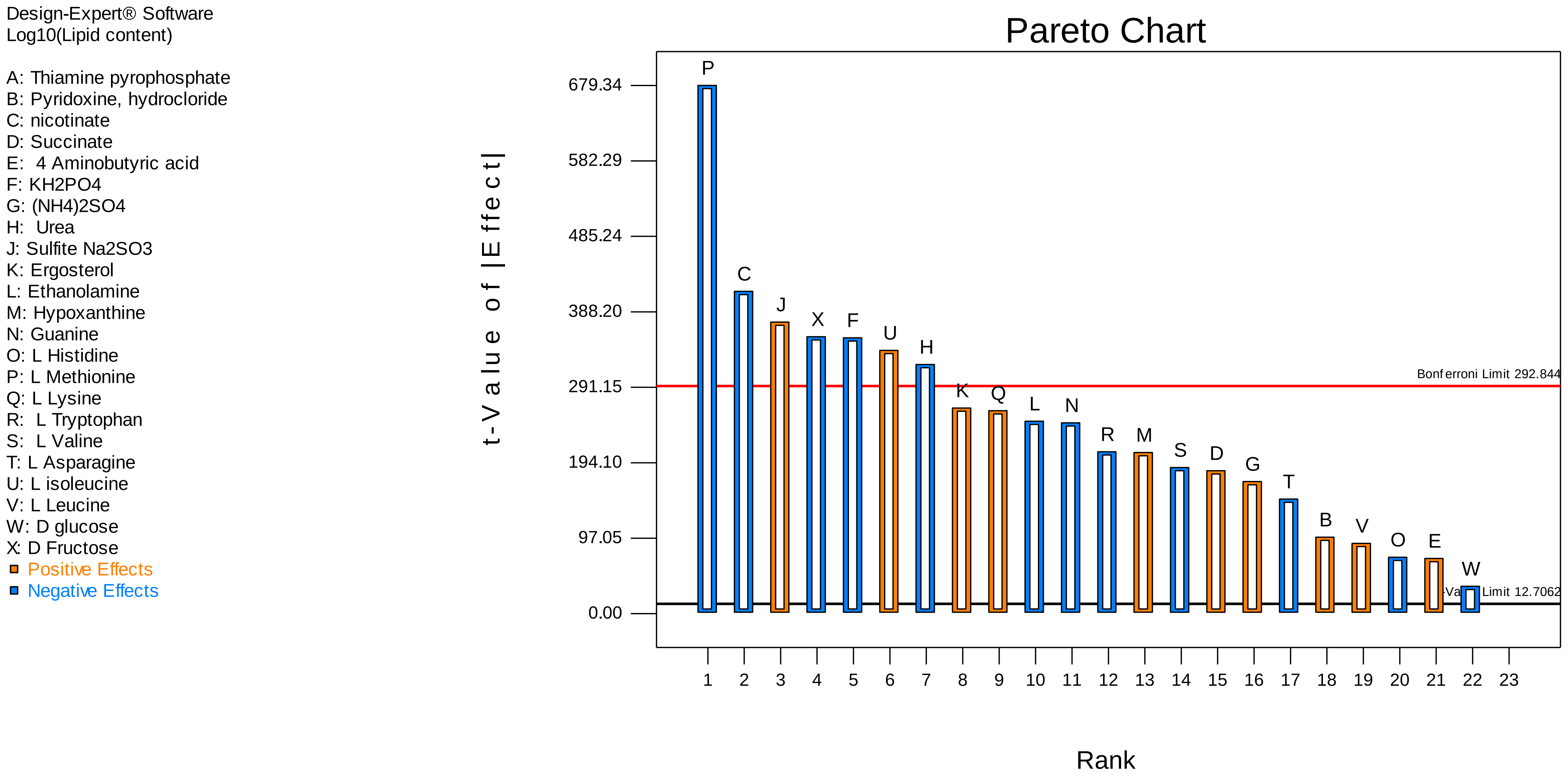

Fig S8: Pareto chart of the effects of chemical compounds predicted by flux balance analysis (FBA) on lipid content of the engineered strain Po1f-1312E+1269IB

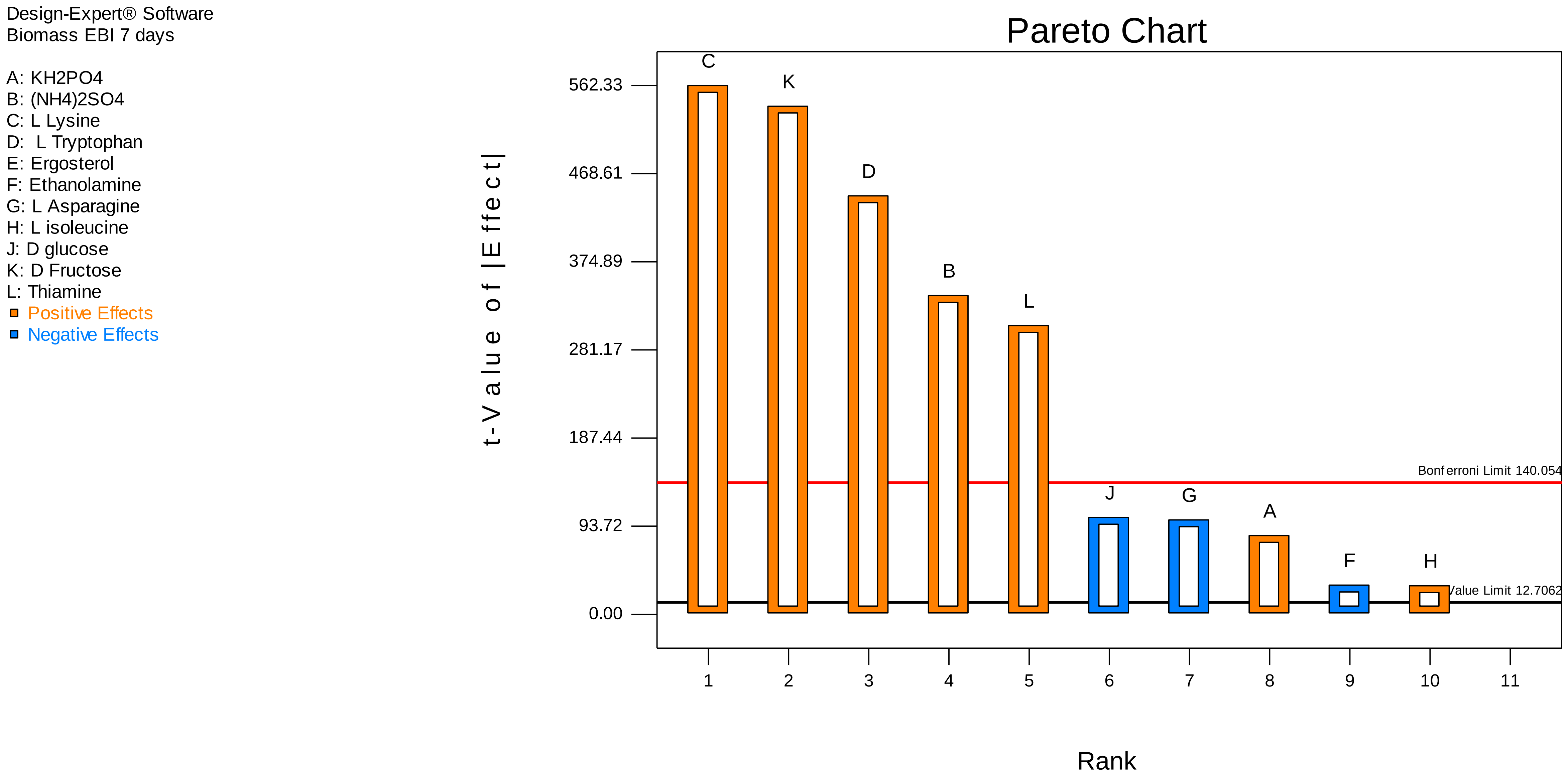

Fig S9: Pareto chart of the effects of chemical compounds predicted by flux balance analysis (FBA) on Biomass production by the engineered strain Po1f-1312E+1269IB in the second tour PBD

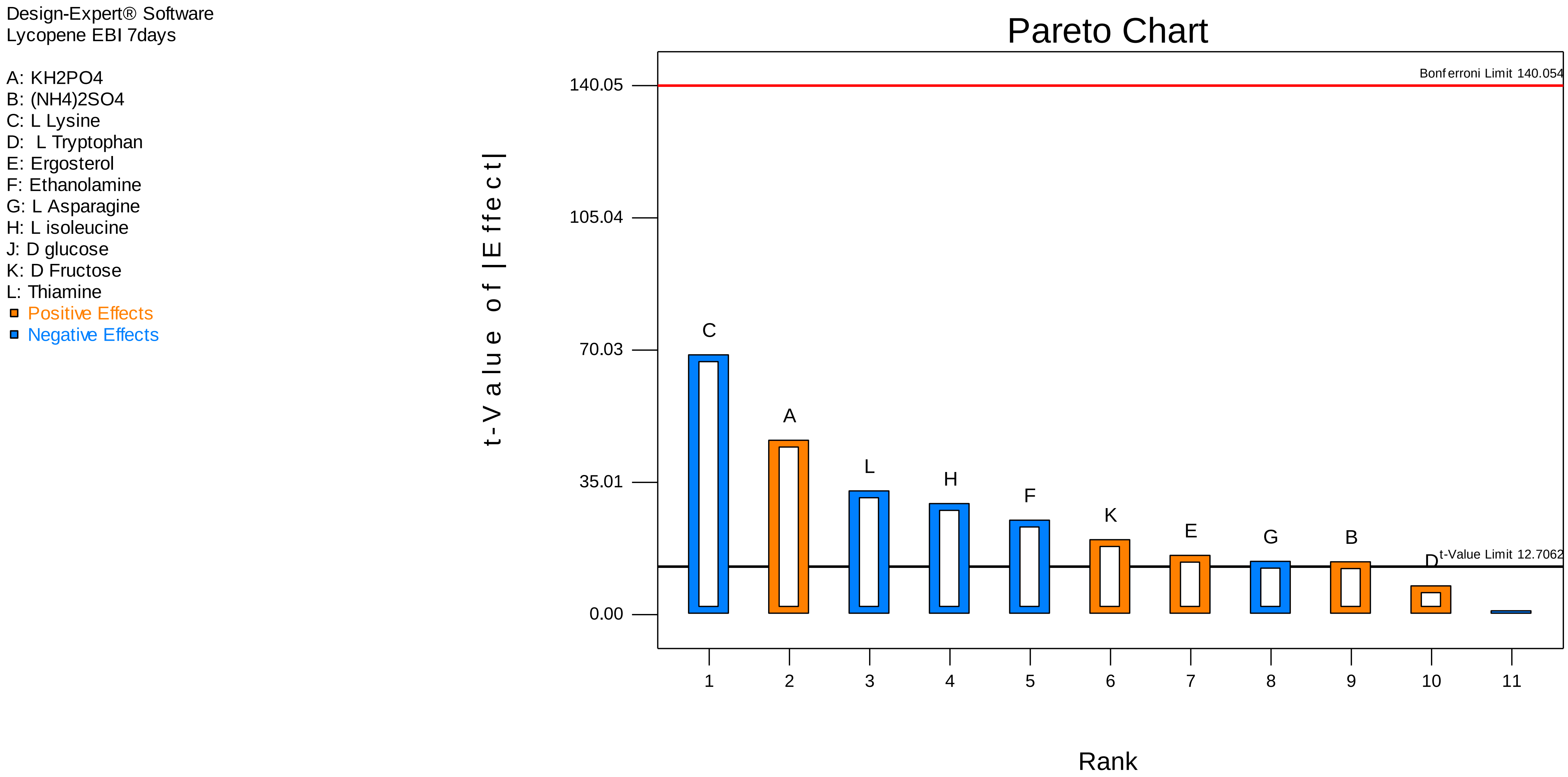

Fig S10: Pareto chart of the effects of chemical compounds predicted by flux balance analysis (FBA) on lycopene production of the engineered strain Po1f-1312E+1269IB in the second tour PBD

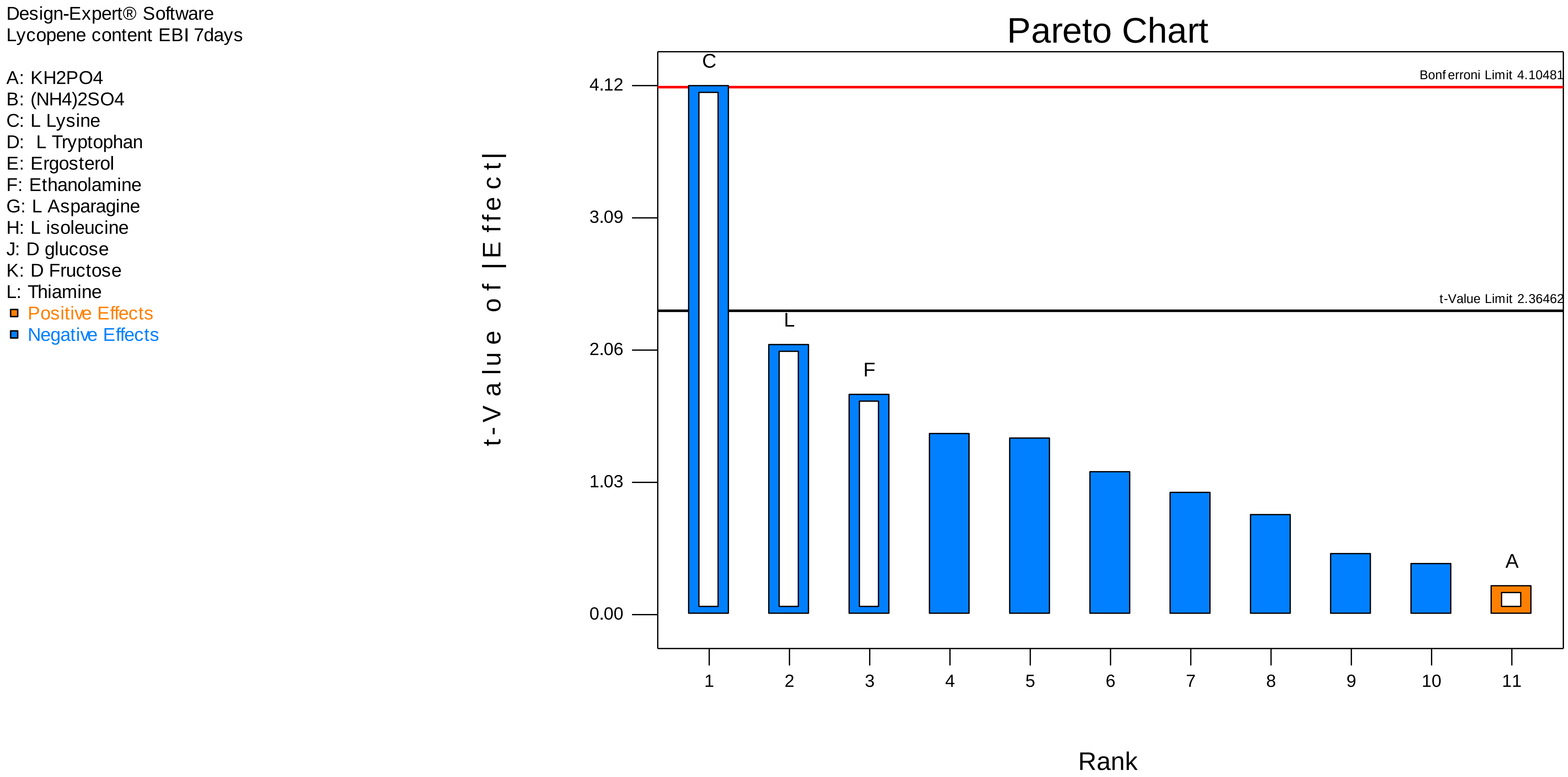

Fig S11: Pareto chart of the effects of chemical compounds predicted by flux balance analysis (FBA) on lycopene content of the engineered strain Po1f-1312E+1269IB in the second tour PBD

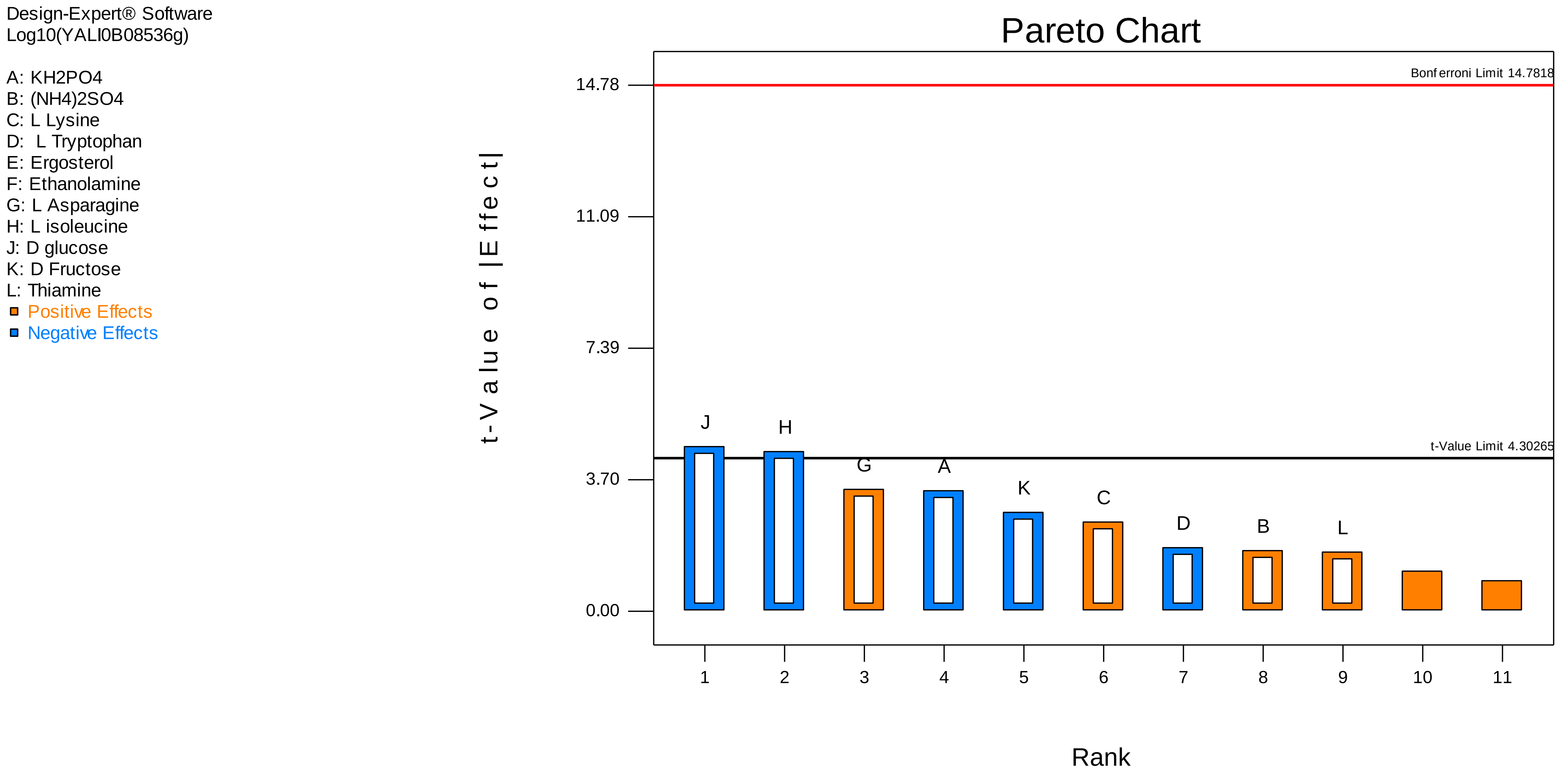

Fig S12: Pareto chart of the effects of chemical compounds predicted by flux balance analysis (FBA) on the expression level of YALI0B08536g in the engineered strain Po1f-1312E+1269IB in the second tour PBD

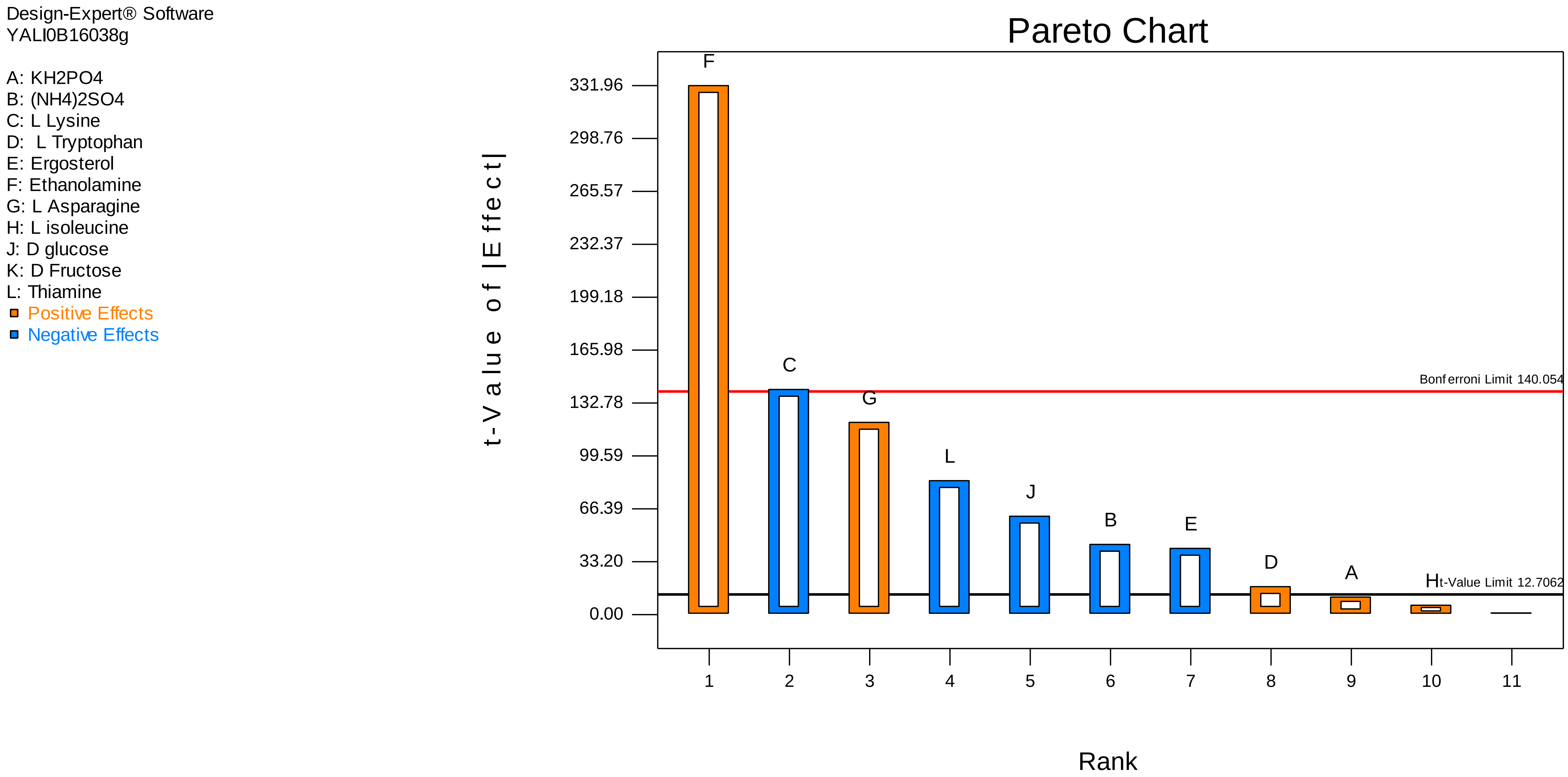

Fig S13: Pareto chart of the effects of chemical compounds predicted by flux balance analysis (FBA) on the expression level of YALI0B16038g in the engineered strain Po1f-1312E+1269IB in the second tour PBD

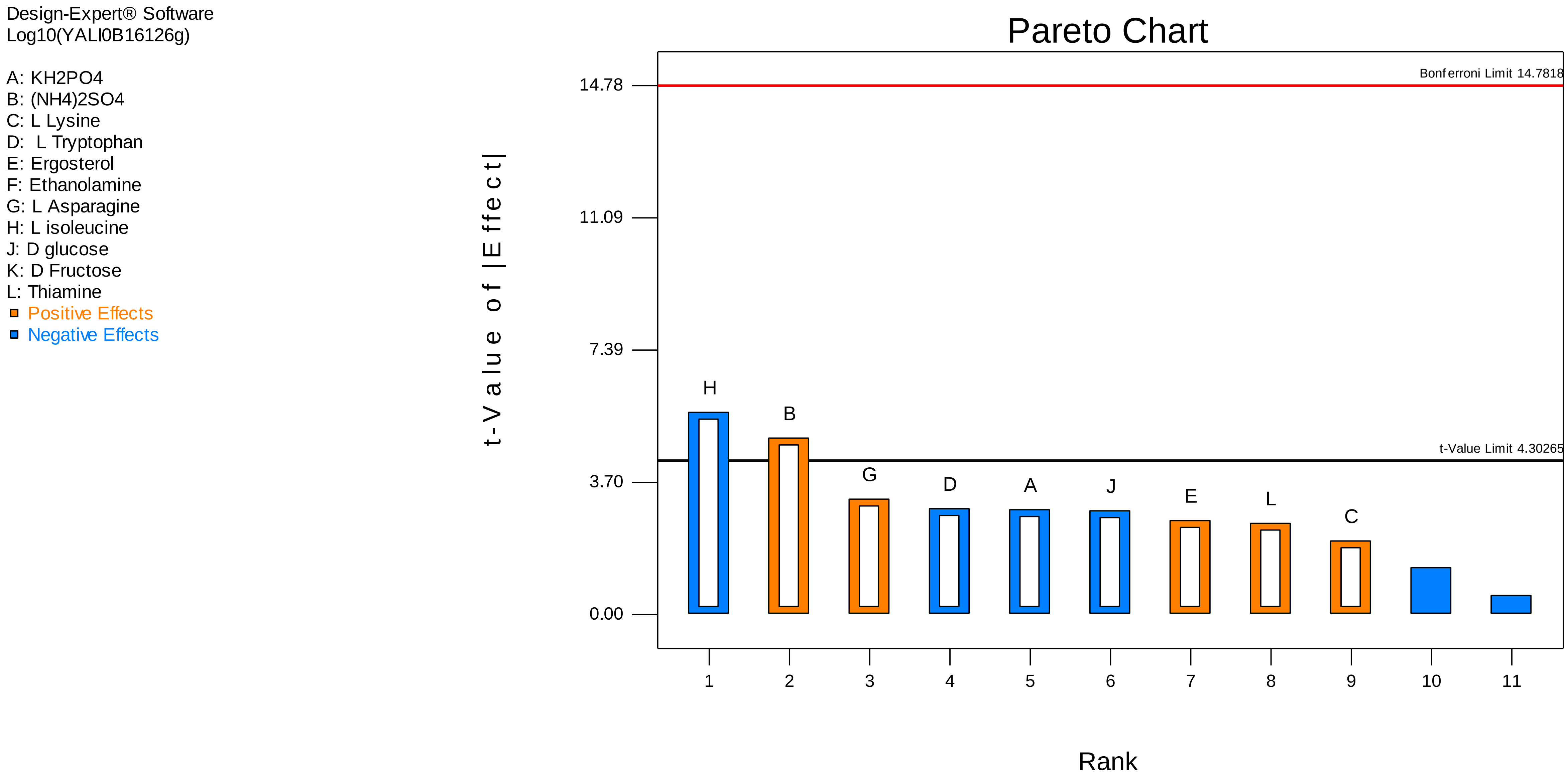

Fig S14: Pareto chart of the effects of chemical compounds predicted by flux balance analysis (FBA) on the expression level of YALI0B16126g in the engineered strain Po1f-1312E+1269IB in the second tour PBD

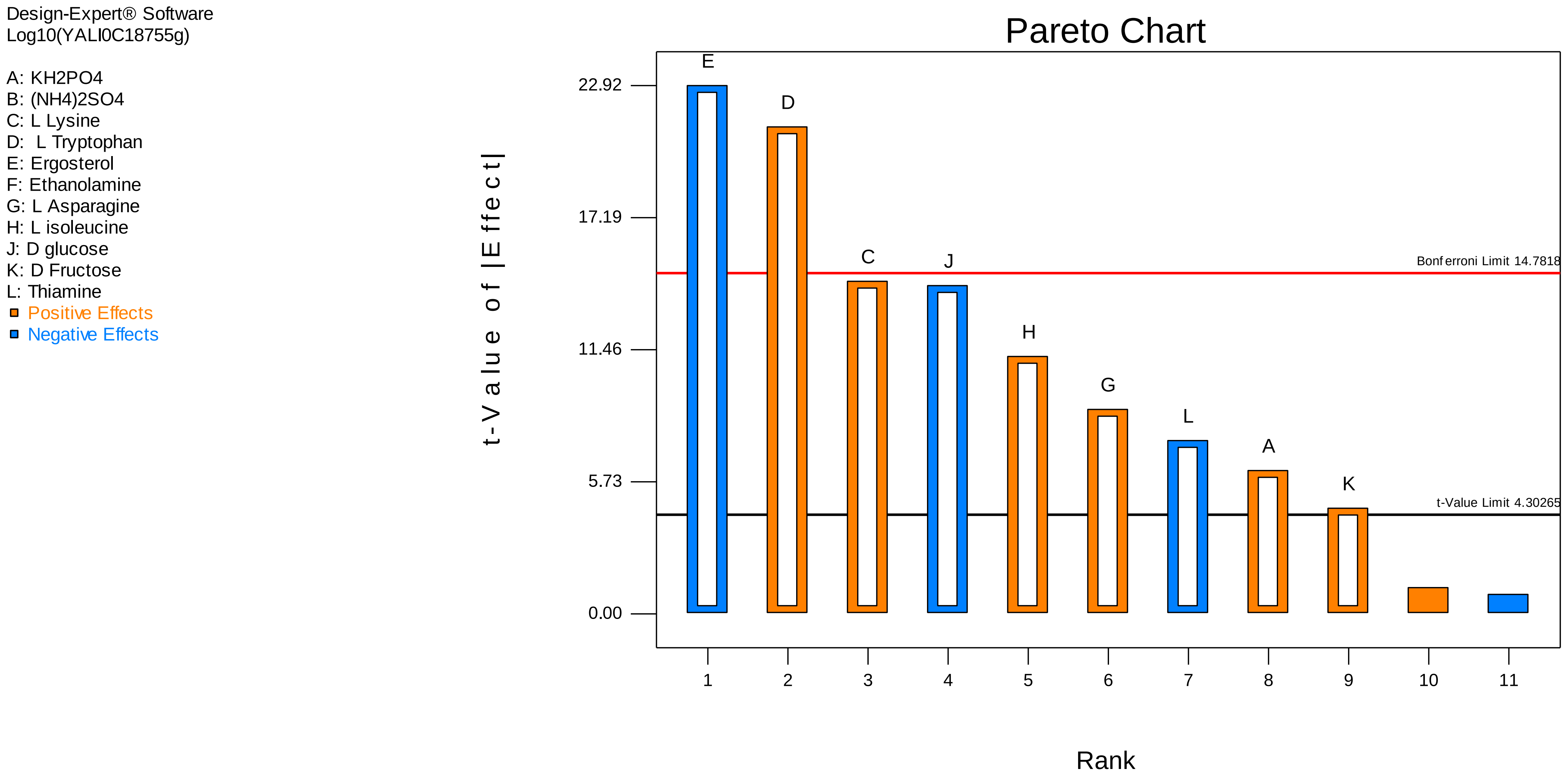

Fig S15: Pareto chart of the effects of chemical compounds predicted by flux balance analysis (FBA) on the expression level of YALI0C18755g in the engineered strain Po1f-1312E+1269IB in the second tour PBD

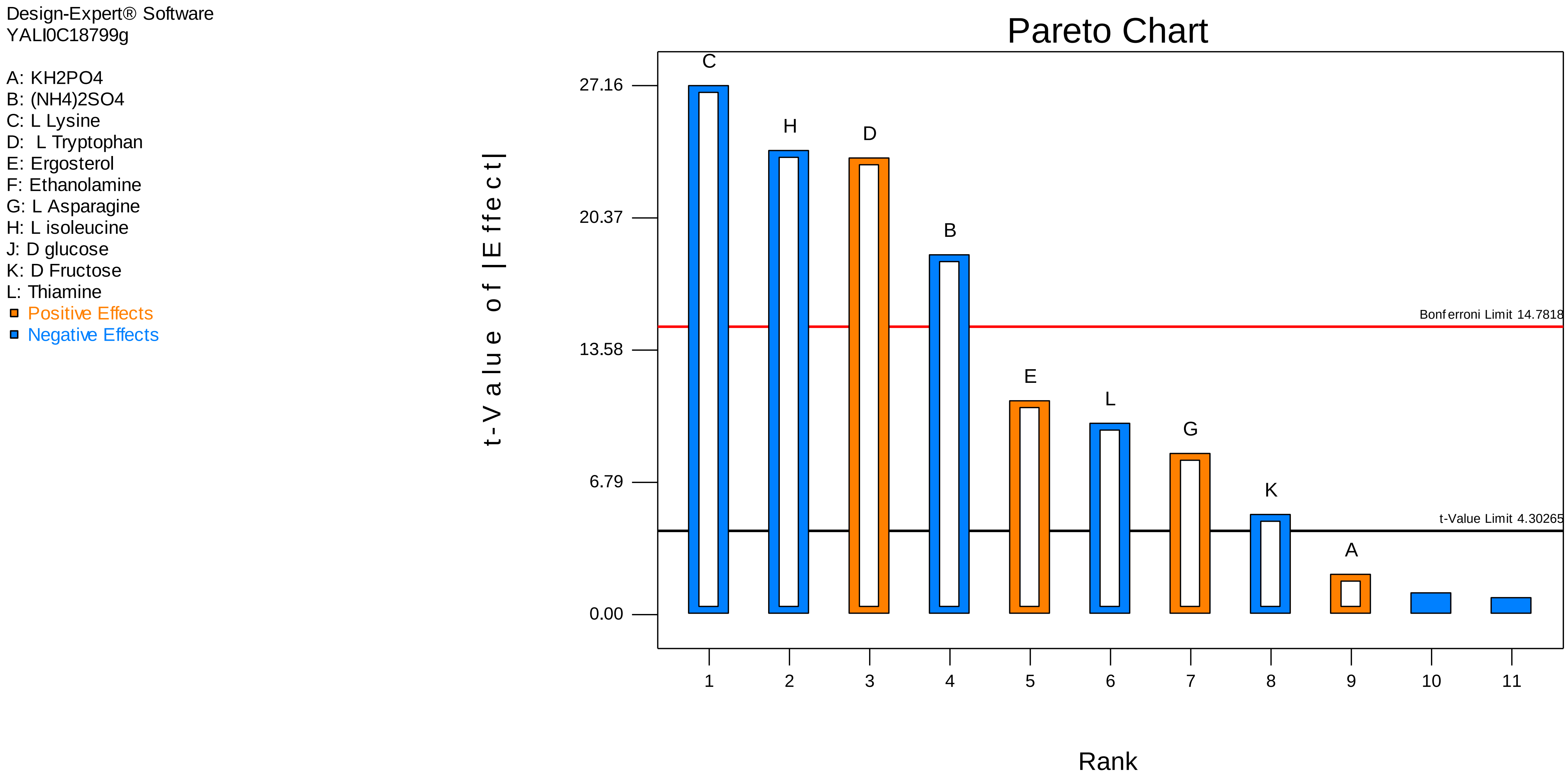

Fig S16: Pareto chart of the effects of chemical compounds predicted by flux balance analysis (FBA) on the expression level of YALI0C18799g in the engineered strain Po1f-1312E+1269IB in the second tour PBD

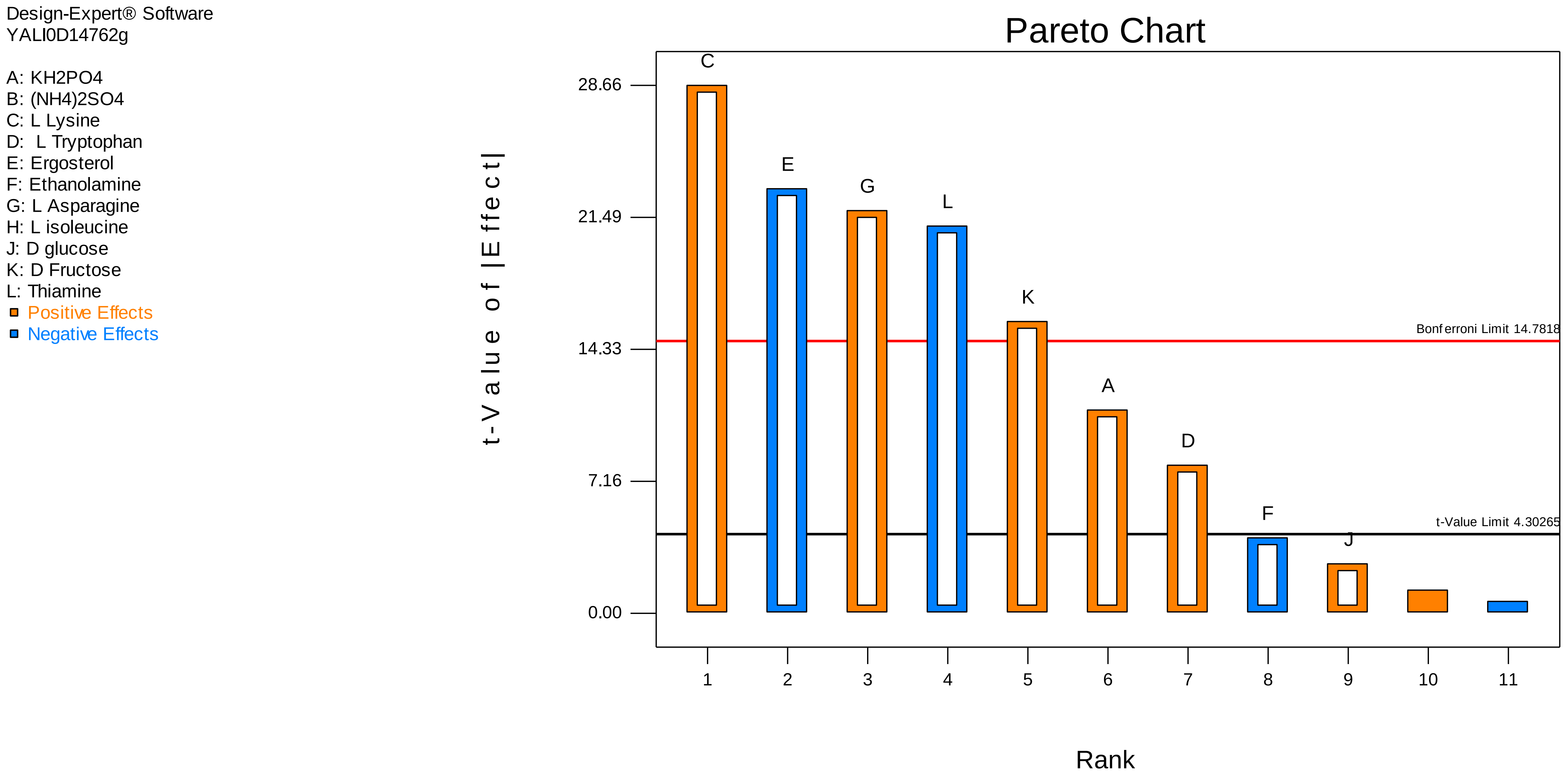

Fig S17: Pareto chart of the effects of chemical compounds predicted by flux balance analysis (FBA) on the expression level of YALI0D14762g in the engineered strain Po1f-1312E+1269IB in the second tour PBD

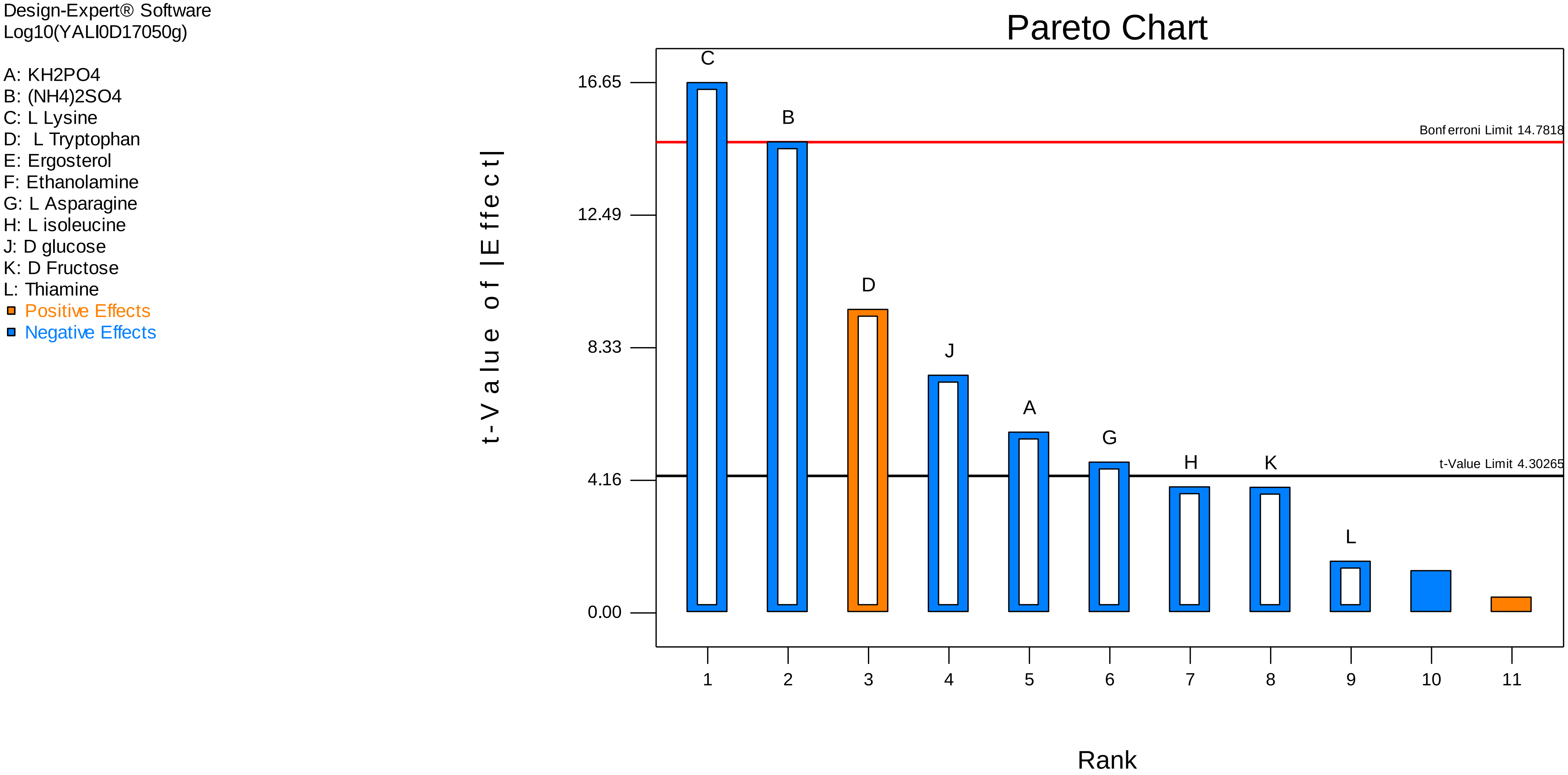

Fig S18: Pareto chart of the effects of chemical compounds predicted by flux balance analysis (FBA) on the expression level of YALI0D17050g in the engineered strain Po1f-1312E+1269IB in the second tour PBD

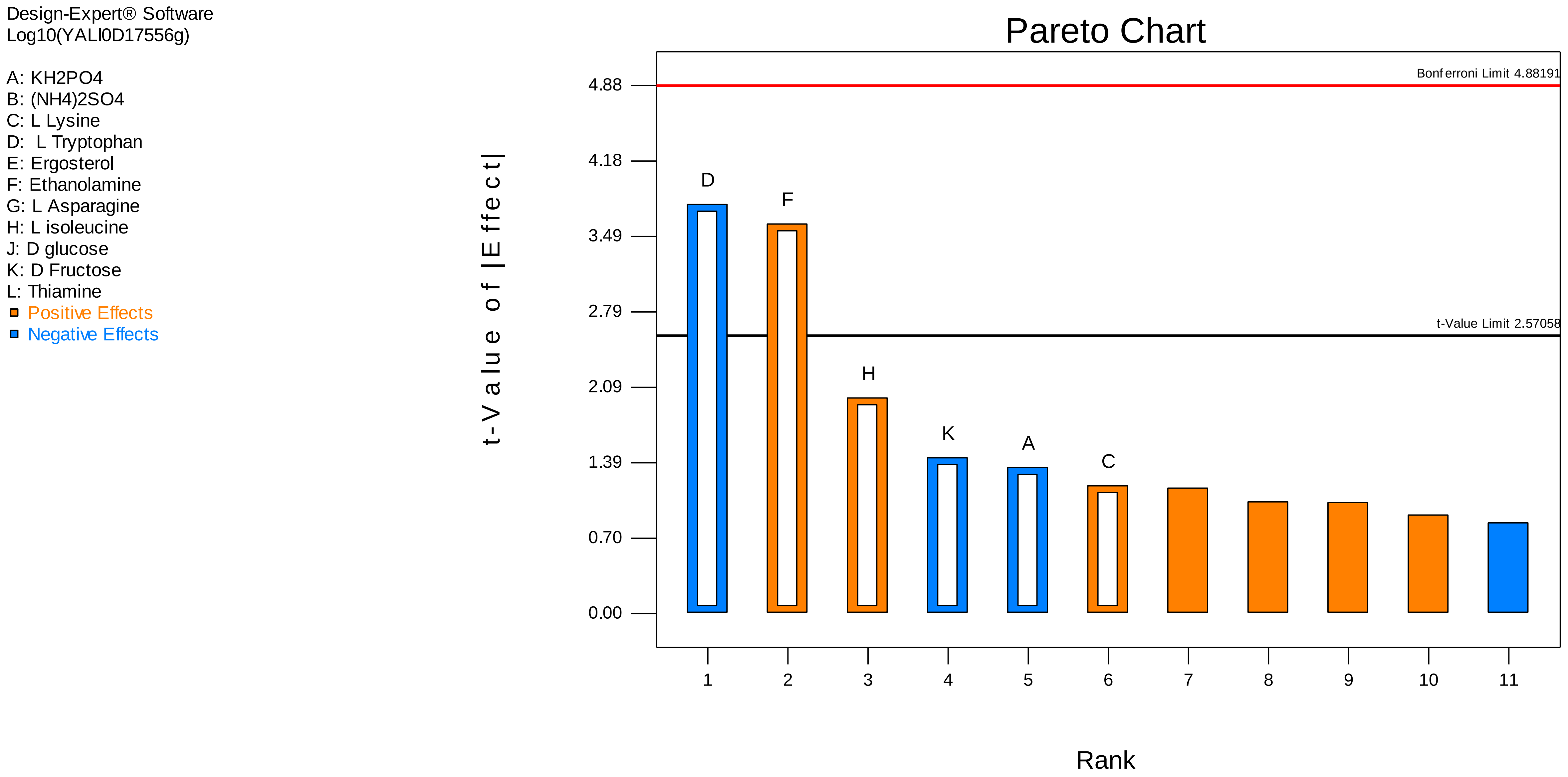

Fig S19: Pareto chart of the effects of chemical compounds predicted by flux balance analysis (FBA) on the expression level of YALI0D17556g in the engineered strain Po1f-1312E+1269IB in the second tour PBD

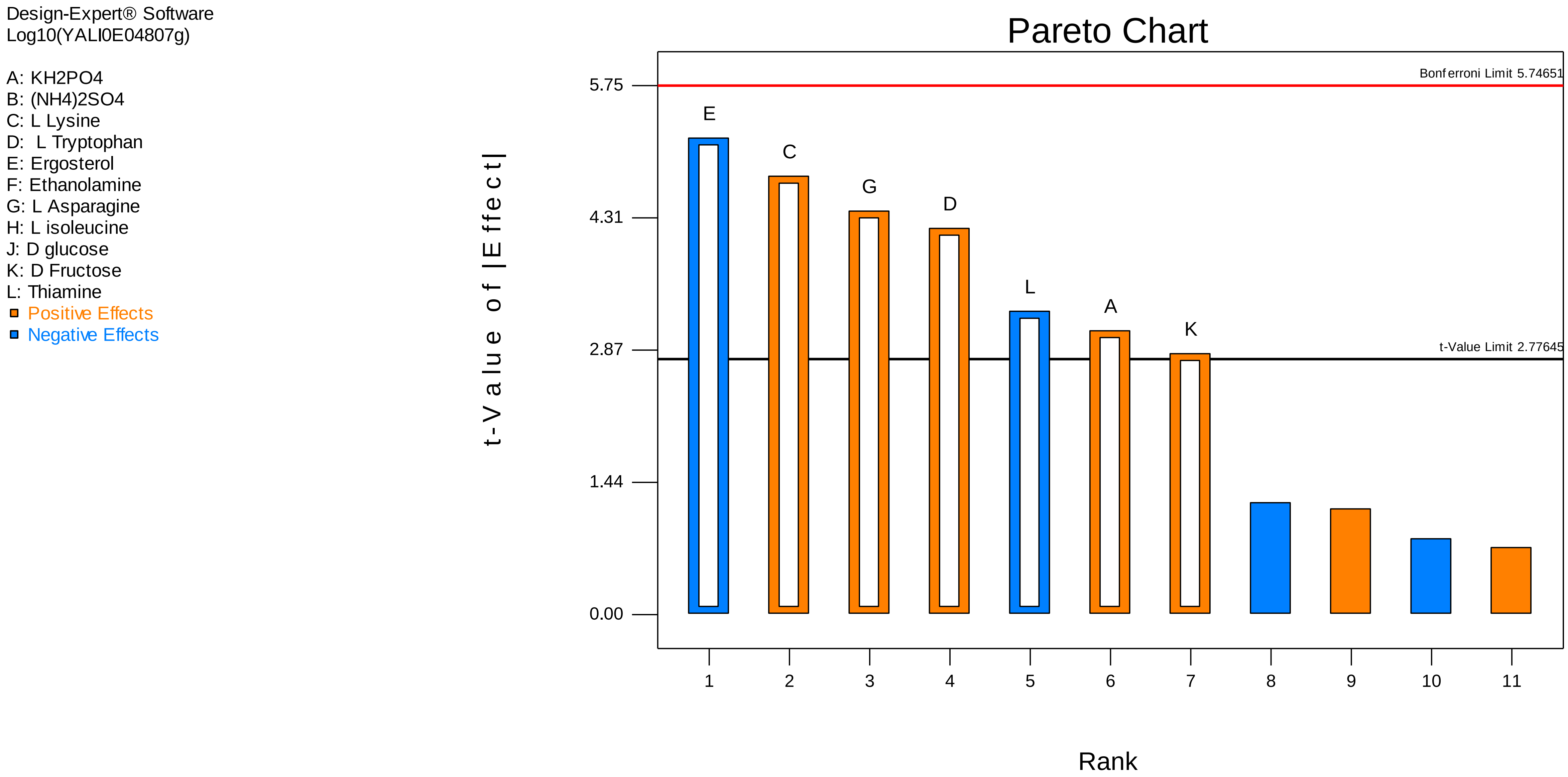

Fig S20: Pareto chart of the effects of chemical compounds predicted by flux balance analysis (FBA) on the expression level of YALI0E04807g in the engineered strain Po1f-1312E+1269IB in the second tour PBD

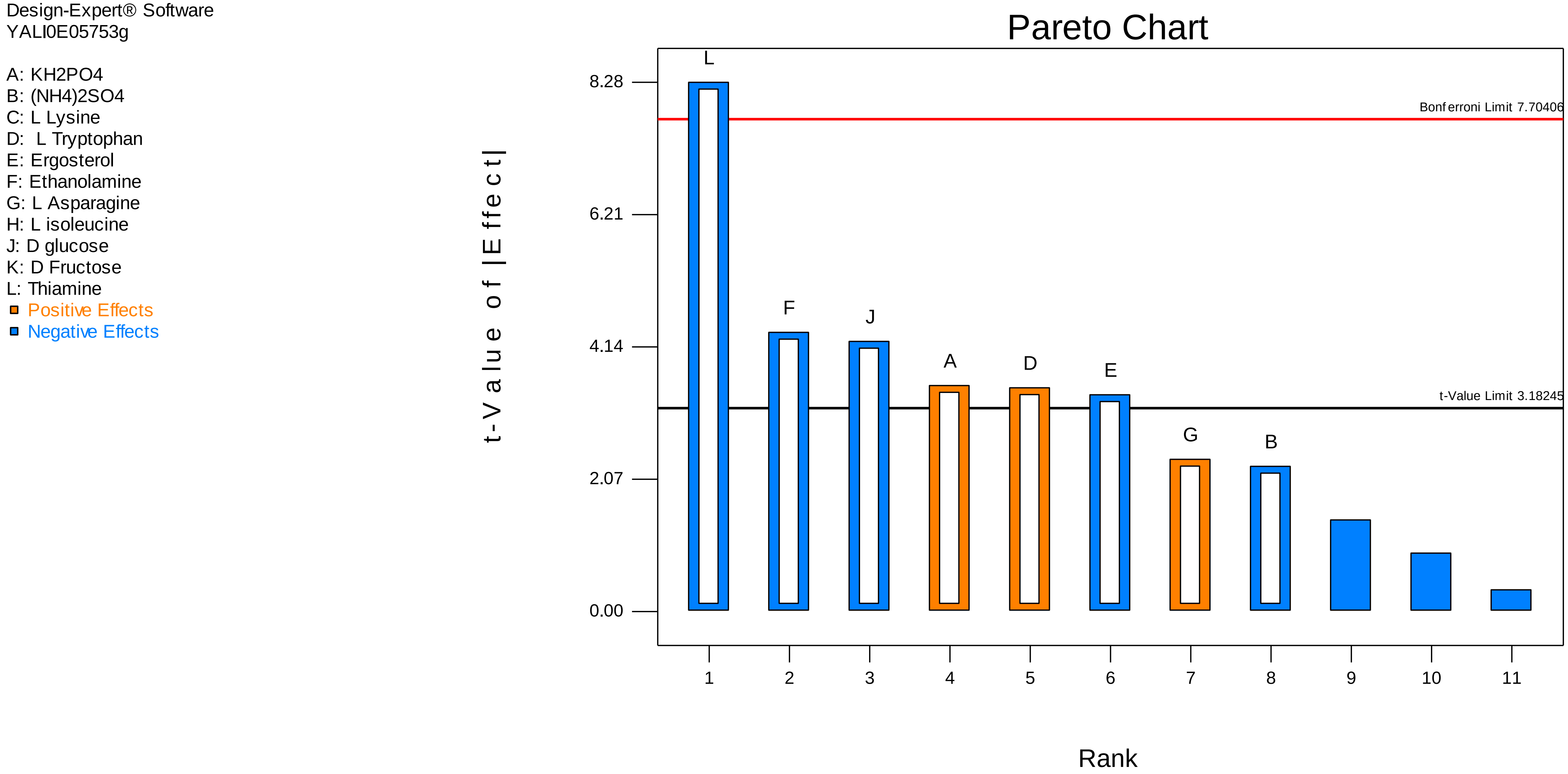

Fig S21: Pareto chart of the effects of chemical compounds predicted by flux balance analysis (FBA) on the expression level of YALI0E05753g in the engineered strain Po1f-1312E+1269IB in the second tour PBD

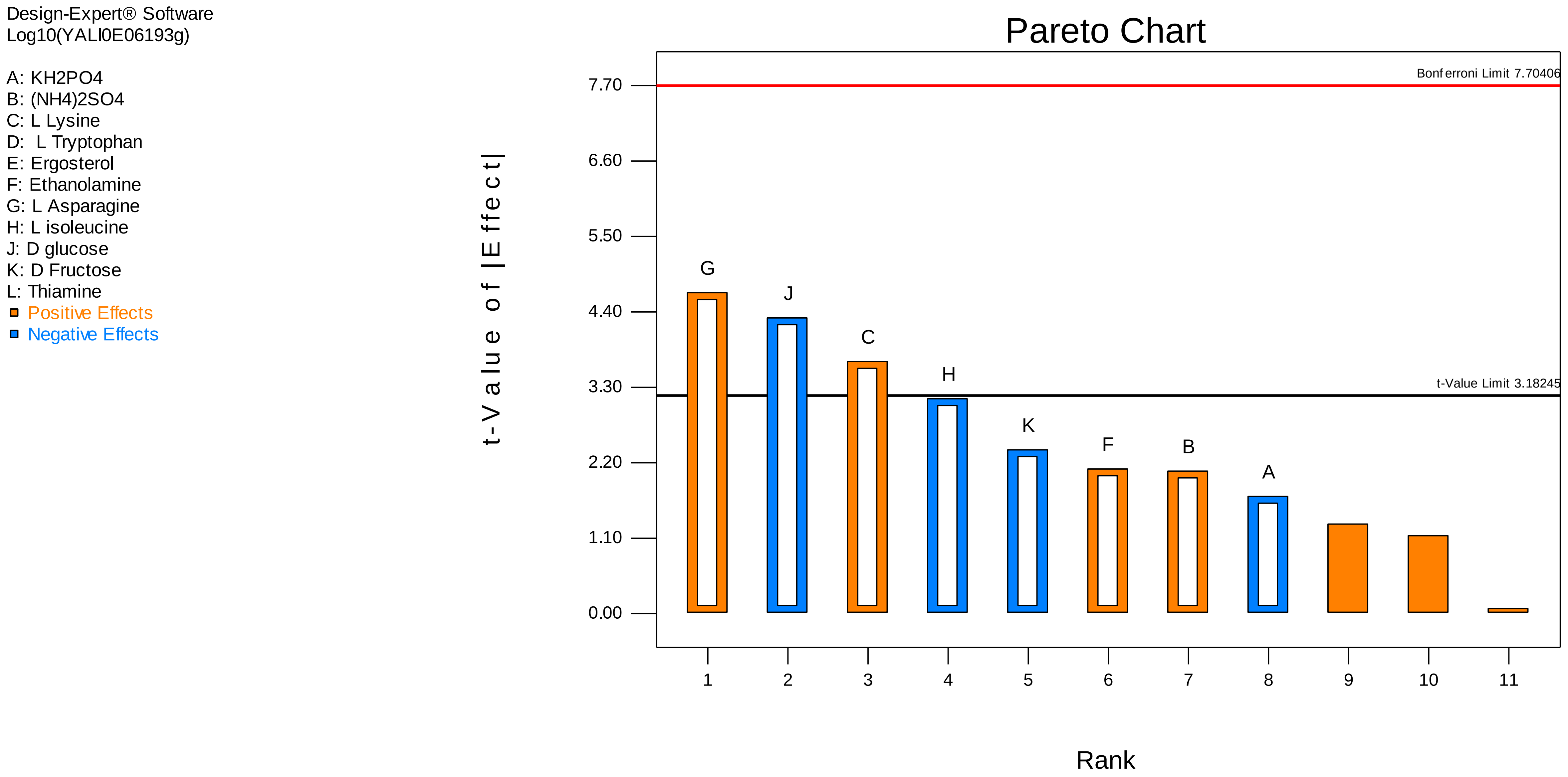

Fig S22: Pareto chart of the effects of chemical compounds predicted by flux balance analysis (FBA) on the expression level of YALI0E06193g in the engineered strain Po1f-1312E+1269IB in the second tour PBD

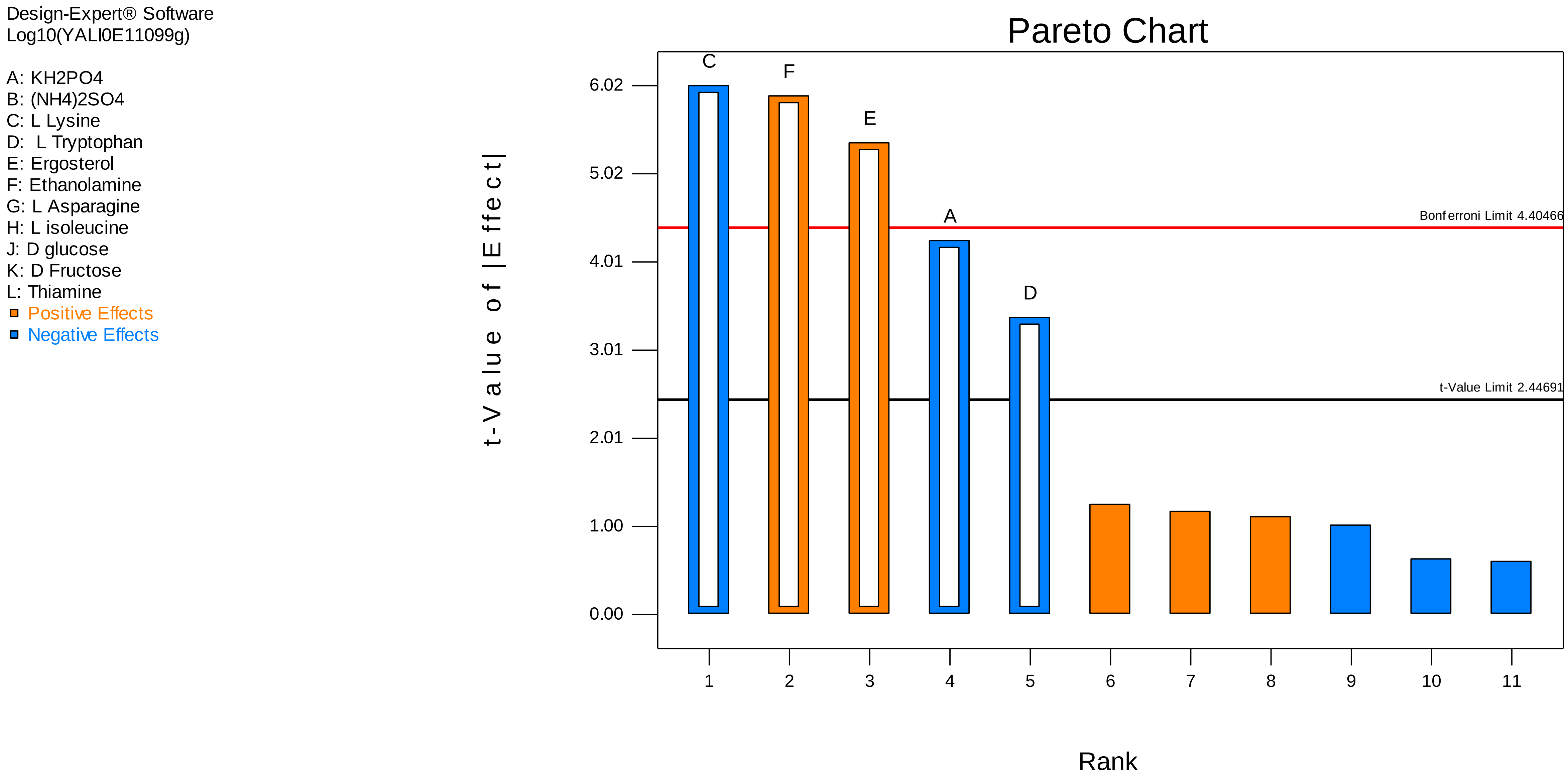

Fig S23: Pareto chart of the effects of chemical compounds predicted by flux balance analysis (FBA) on the expression level of YALI0E11099g in the engineered strain Po1f-1312E+1269IB in the second tour PBD

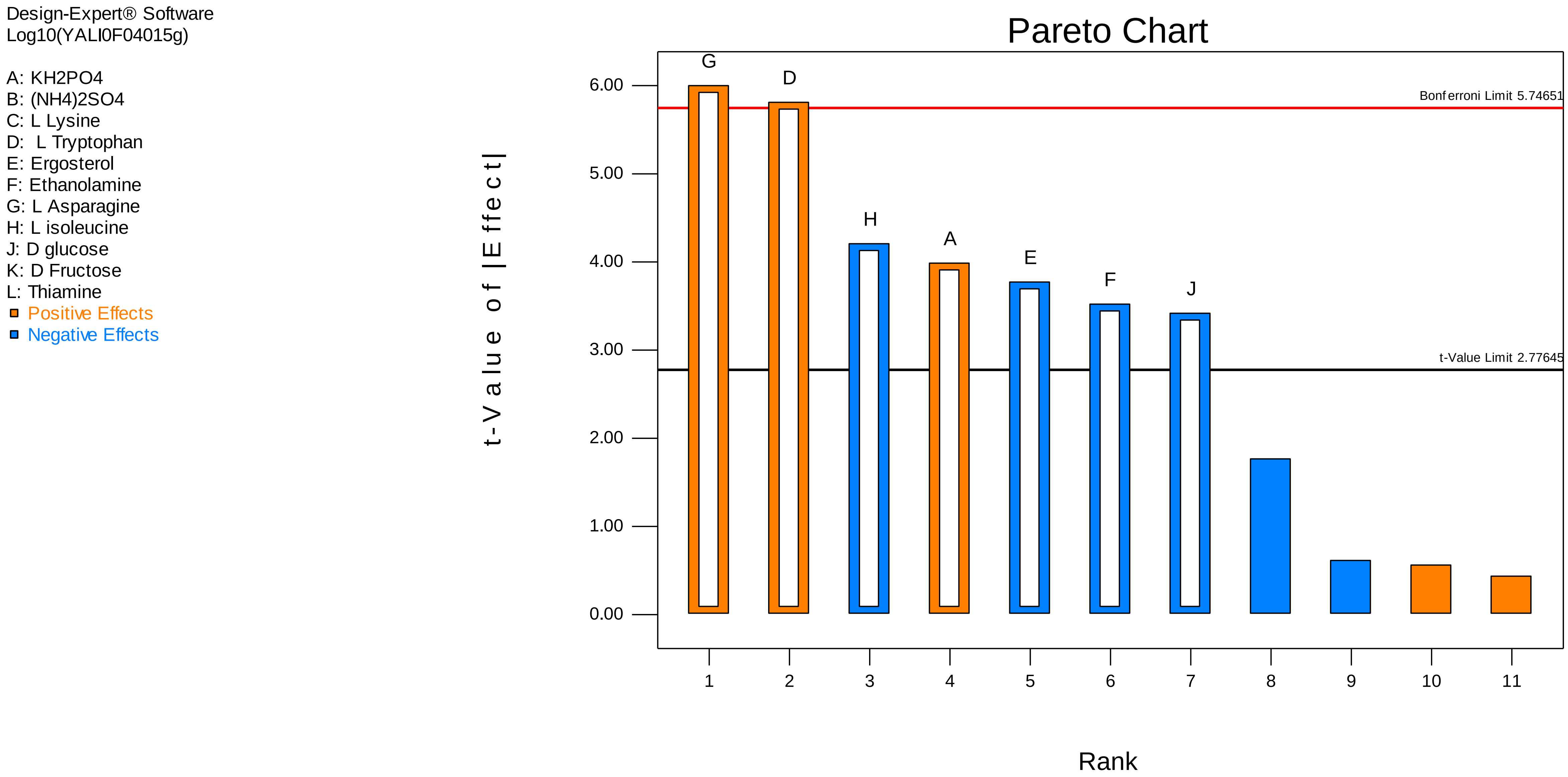

Fig S24: Pareto chart of the effects of chemical compounds predicted by flux balance analysis (FBA) on the expression level of YALI0F04015g in the engineered strain Po1f-1312E+1269IB in the second tour PBD

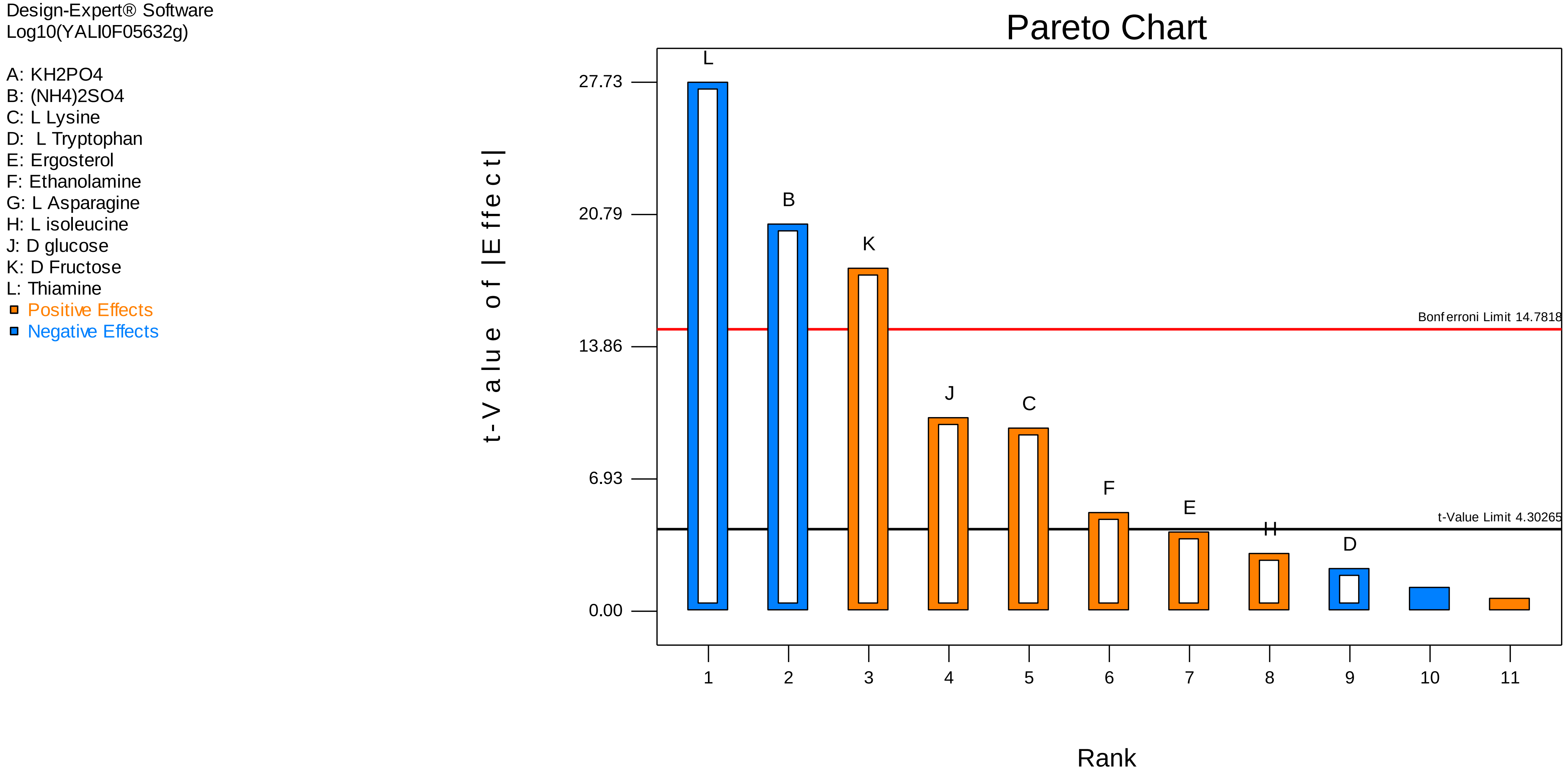

Fig S25: Pareto chart of the effects of chemical compounds predicted by flux balance analysis (FBA) on the expression level of YALI0F05632g the engineered strain Po1f-1312E+1269IB in the second tour PBD

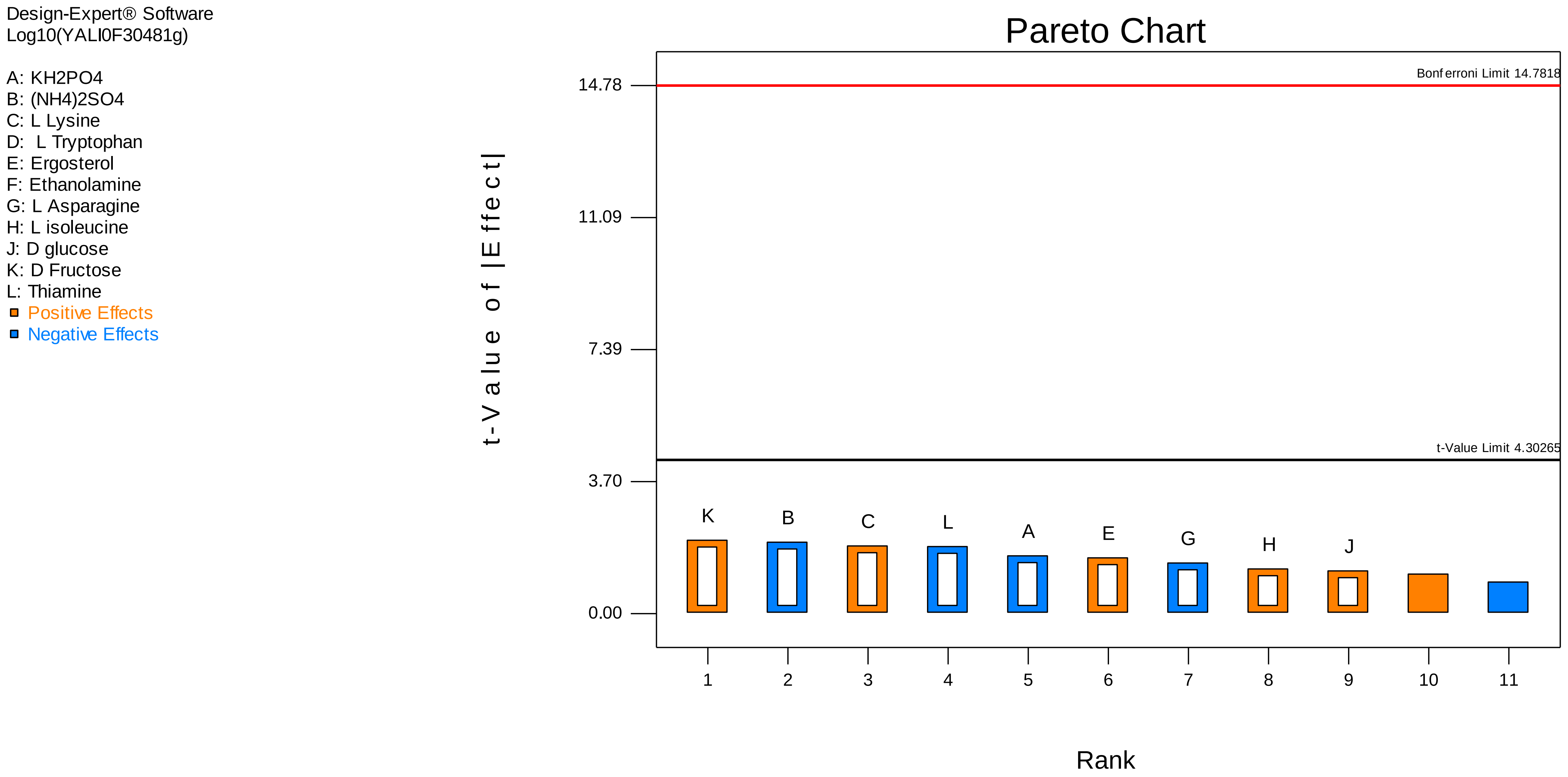

Fig S26: Pareto chart of the effects of chemical compounds predicted by flux balance analysis (FBA) on the expression level of YALI0F30481g in the engineered strain Po1f-1312E+1269IB in the second tour PBD

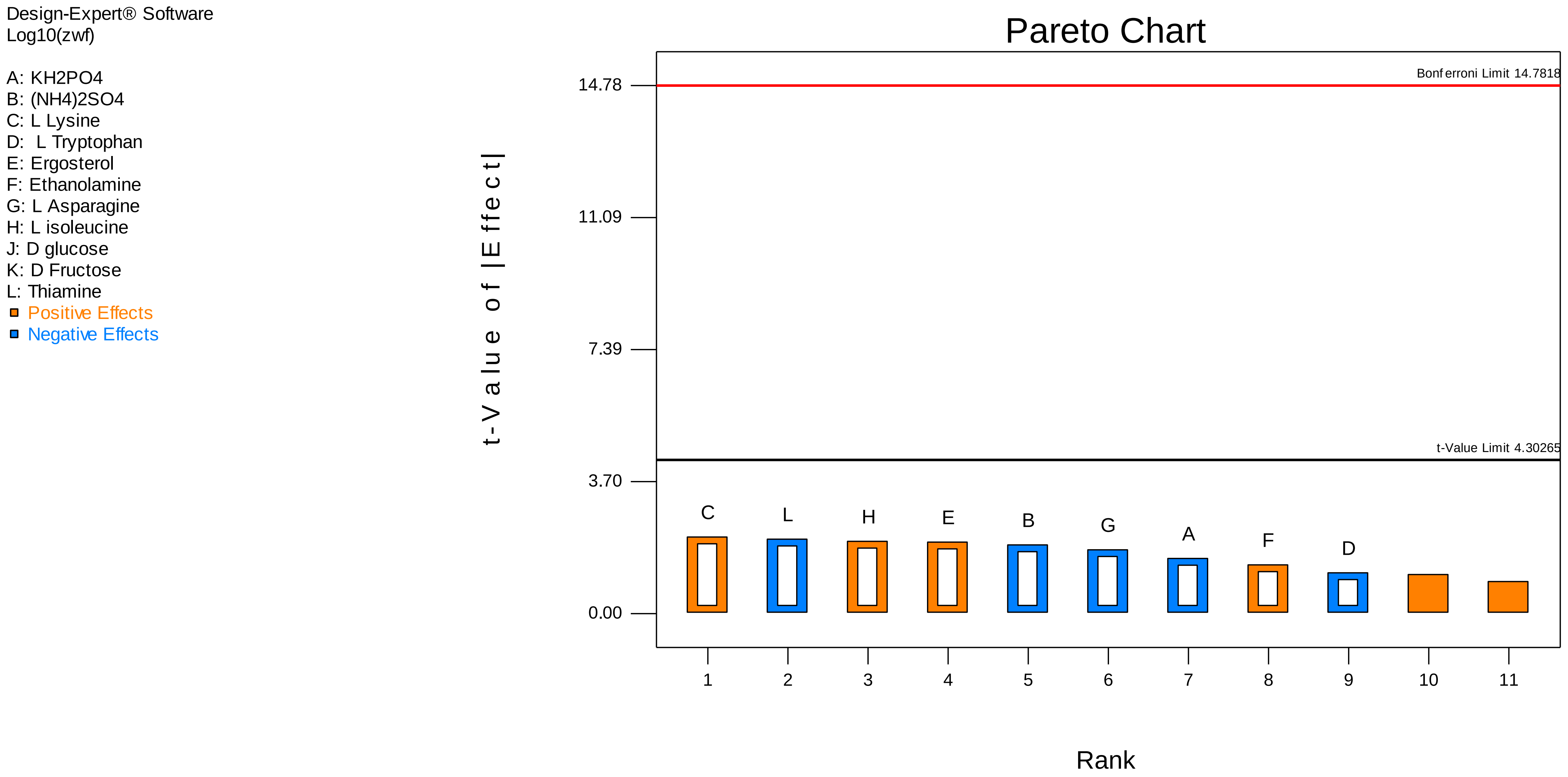

Fig S27: Pareto chart of the effects of chemical compounds predicted by flux balance analysis (FBA) on the expression level of zwf in the engineered strain Po1f-1312E+1269IB in the second tour PBD

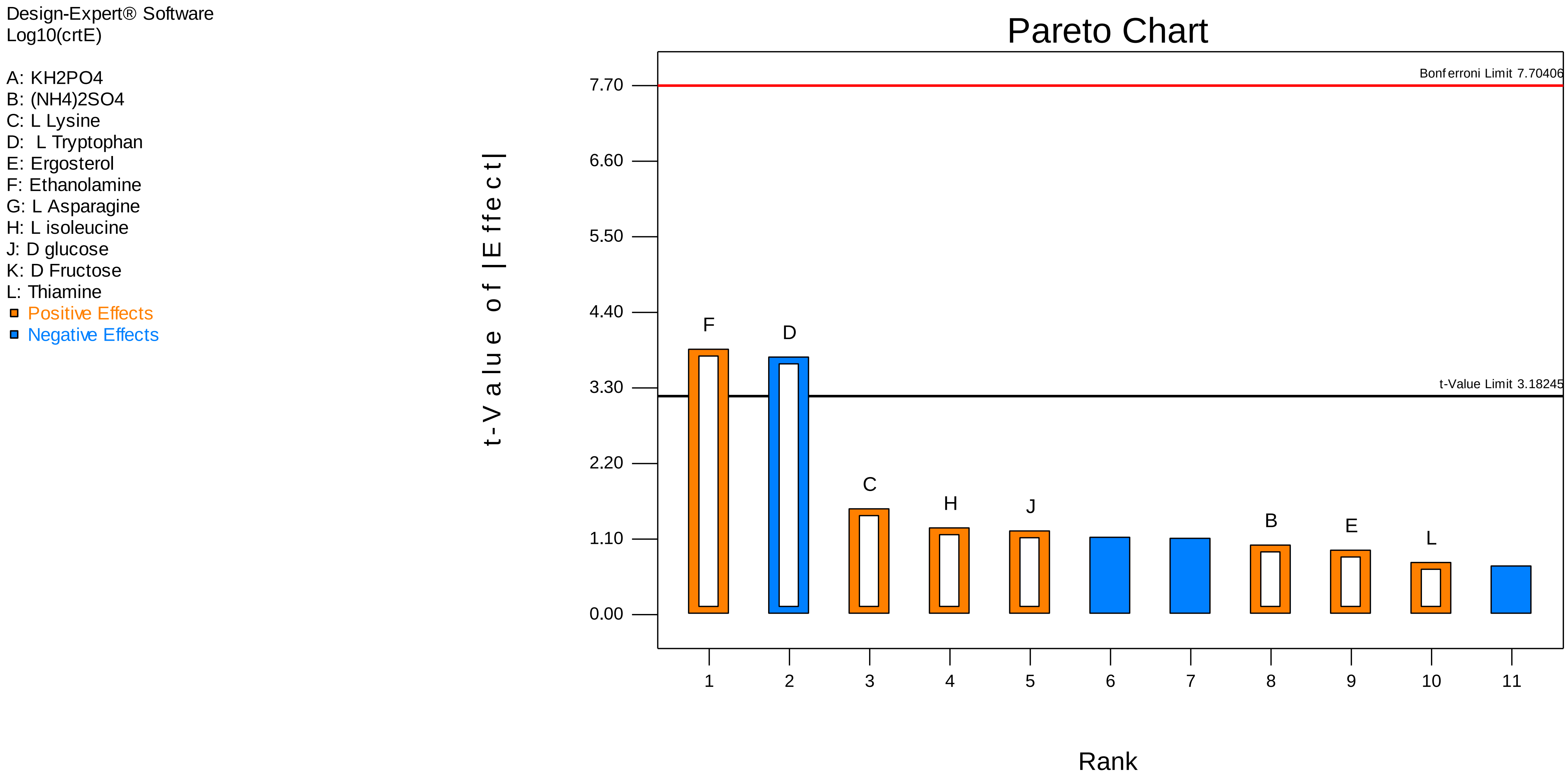

Fig S28: Pareto chart of the effects of chemical compounds predicted by flux balance analysis (FBA) on the expression level of crtE in the engineered strain Po1f-1312E+1269IB in the second tour PBD

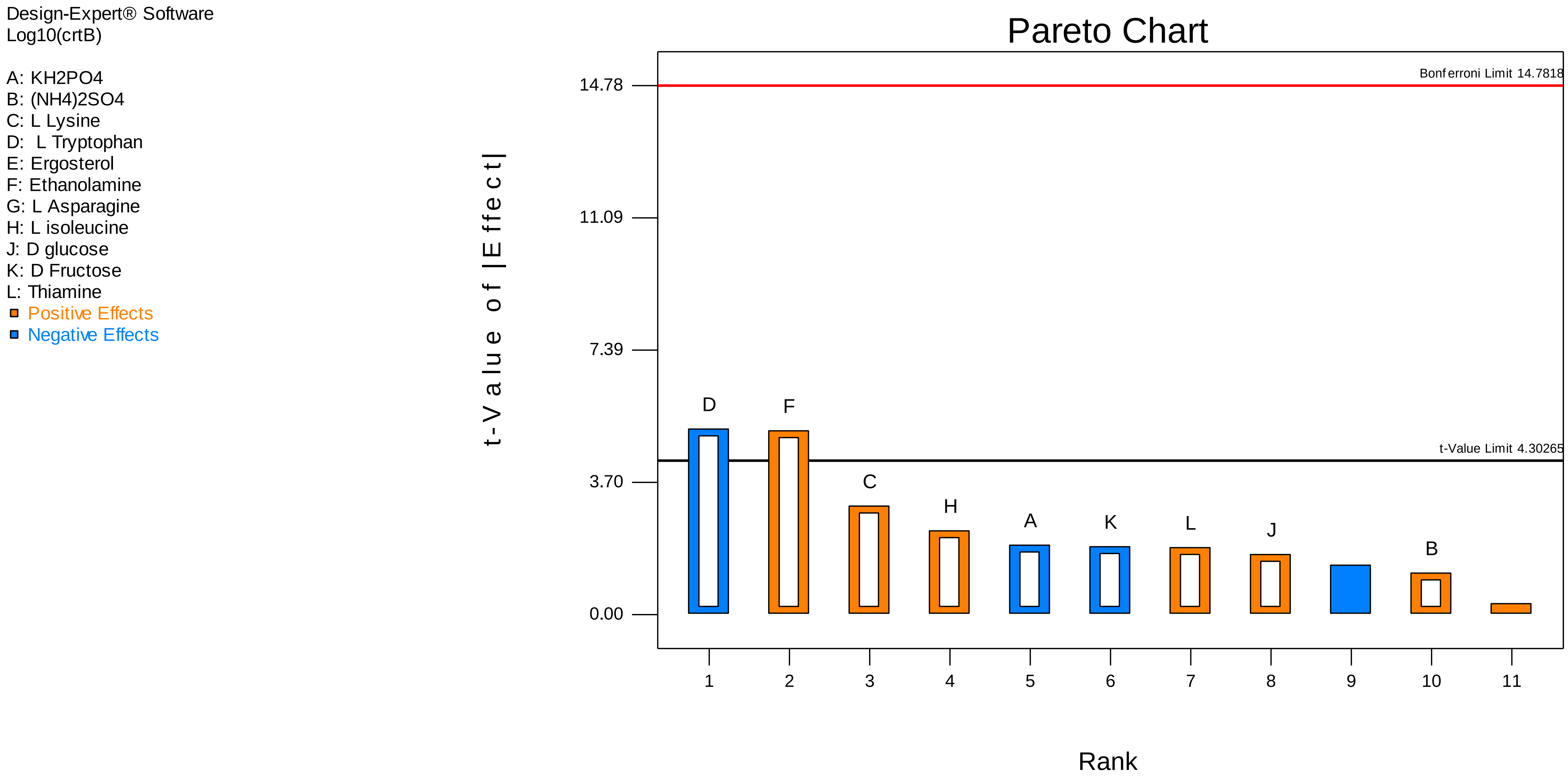

Fig S29: Pareto chart of the effects of chemical compounds predicted by flux balance analysis (FBA) on the expression level of crtB in the engineered strain Po1f-1312E+1269IB in the second tour PBD

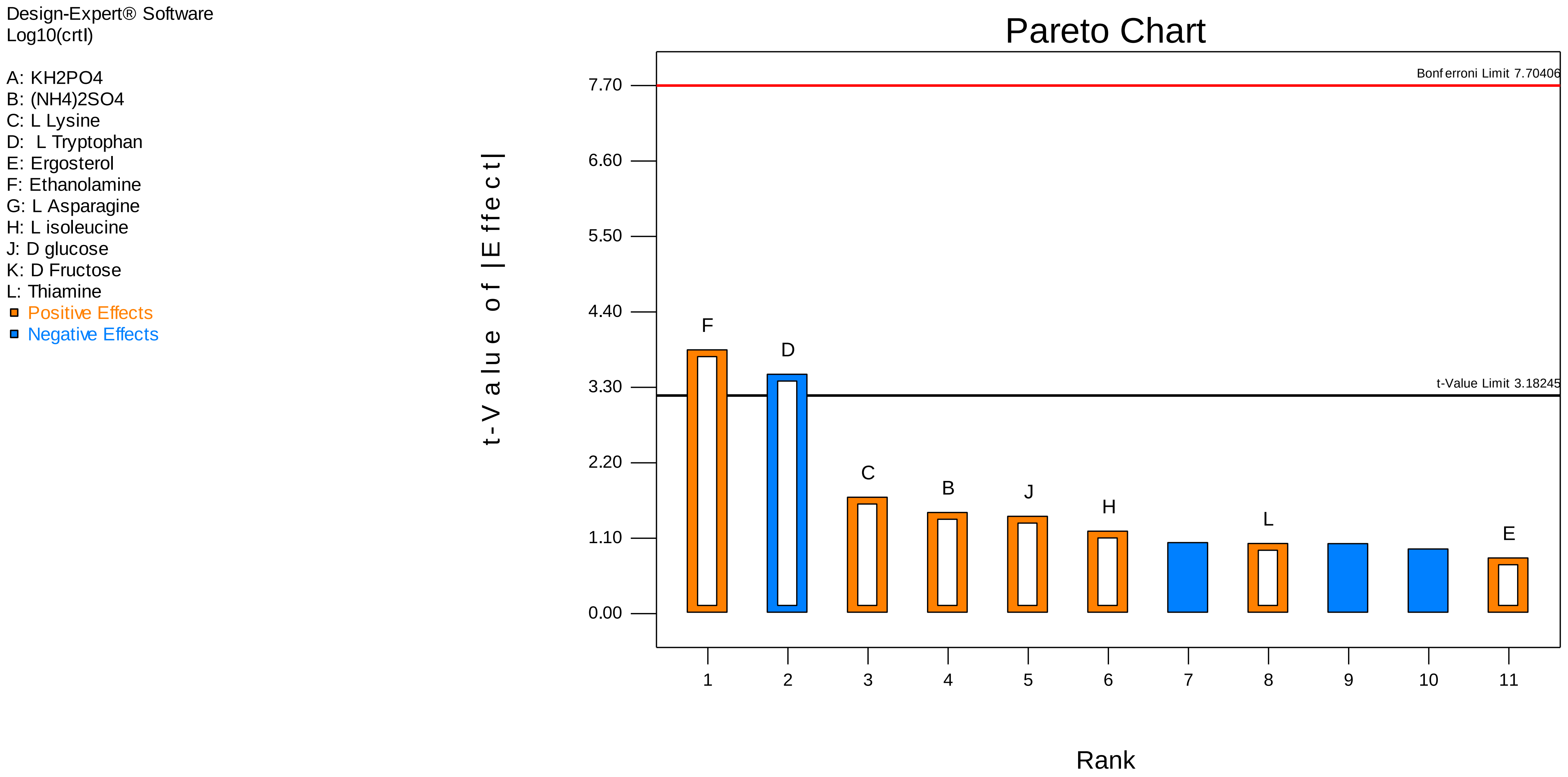

Fig S30: Pareto chart of the effects of chemical compounds predicted by flux balance analysis (FBA) on the expression level of crtI in the engineered strain Po1f-1312E+1269IB in the second tour PBD
