# Supplementary material for: Flux Balance Analysis Inspired Bioprocess Upgrading for Lycopene Production by a Metabolically Engineered Strain of Yarrowia lipolytica
Source: Metabolites. 2015 Dec 21;5(4):794–813. doi: 10.3390/metabo5040794 (PMC4693195; doi:10.3390/metabo5040794)
Supplement: Supplementary File 1 [file metabolites-05-00794-s001.zip › metabolites-99322-supplementary-final/Supplementary File 2.pdf]

Supplementary File 2

Model Correction

Genome-scale metabolic model iYL619_PCP of *Y. lipolytica* was completed reconstruction by our laboratory several years ago [1]. The curated model iYL619_PCP was derived from the available knowledge in public databases (such as, KEGG, UniProt, IMG) and scientific publications. However, some imperfections about topological network and setting parameters of model were found gradually to result in simulations far from experiment data. Therefore, we performed model correction based on comparison between simulation and experiment data as follows, and the updated model, model yli v1.7, is available at http://www.echaoceshi.com/ECLOSB/CN/Default.aspx.

Some modifications of programming language of iYL619_PCP, first of all, avoid errors when to read the model using a function of the Cobra Toolbox 2.0 based on Matlab. Since *Y. lipolytica* is a strictly aerobic microorganism [2], however, model iYL619_PCP simulated the specific growth rate of 0.0269 h^{-1} when respectively setting the uptake rates of O_2 and glucose at 0 and 20 mmol/g DCW/h. This unresonable simulation was resolved by turning reaction R0795 into an irreversible one to block autosynthesis of oxygen, *i.e.*, R0795 (pyridoxine + oxygen <=> pyridoxal + hydrogen peroxide) in the original model iYL619_PCP was replaced with an irreversible one (pyridoxine + oxygen => pyridoxal + hydrogen peroxide). To barrier some unreasonable conversion of vital factors, additionally, some other reversible reactions which can be catalyzed by different cofactor-dependent (NAD+/NADH, NADP+/NADPH) enzymes were also turned into irreversible ones, such as R0453 and R0454. Besides, a missing reaction R0378, named pyruvate dehydrogenase complex, was added into model, and R0785 was removed because it was a duplicate of R0381.

In the original model, reaction R0437 was an interregional hydrogen transport reaction, simultaneously co-producing ATP without consuming any substrate. The maximum specific growth rate was 0.0439 h^{-1} when setting glucose uptake rate at 20 mmol/g DCW/h in aerobic condition. Meanwhile, we performed flux variability analysis (FVA) to reaction R0437, and result showed range from 177.005 to 743.942 mmol/g DCW/h, suggesting that at least 177 mmol/g DCW/h ATP was produced without glucose. When tried to delete reaction R0437 (the upper and lower bounds of R0437 were both set at zero), the maximum specific growth rate dropped to 0.0350 h^{-1}, but this specific growth rate is closer to experiment data (Figure S1). As Figure S1 shows, cell only grows under the predicted minimal media and the maximum specific growth rate is 0.0352 h^{-1} [1]. Note that model yli v1.7 and iYL619_PCP are equal in predicting the glucose minimal media (Table S1).

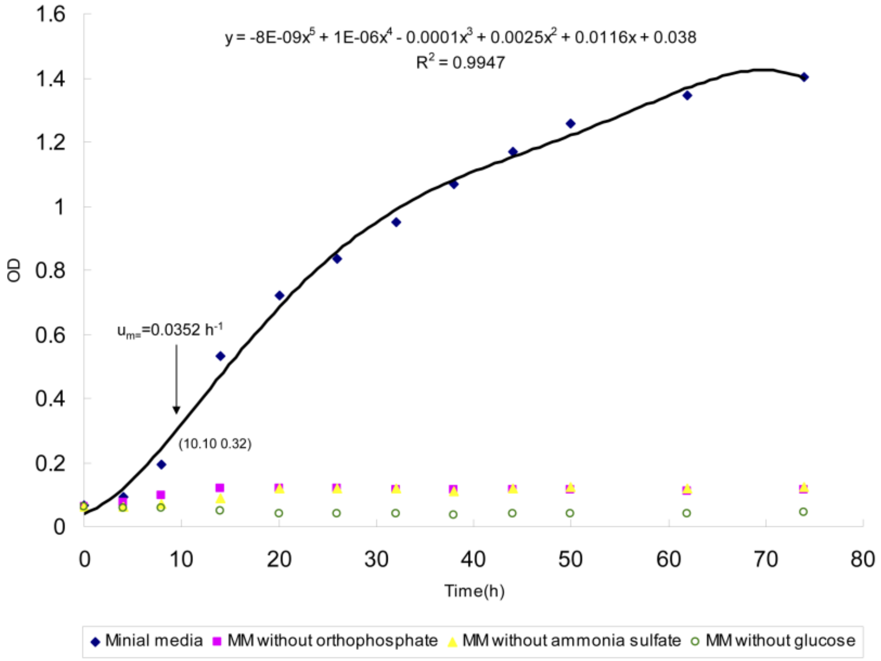

Figure S1. Experimental validation of the minimal media predicted in silico with the model iYL619_PCP. The composition of minimal media in experiment is as follows: glucose (20 g/L), NH_4SO_4 (3 g/L), KH_2PO_4 (2 g/L). The OD of culture media after inoculation was about 0.06. (This figure adopted from reference [1]).

Table S1. The *in silico* glucose minimal media of model iYL619_PCP [1].

ID	Reaction Name	Equation	LB [2]	UB [3]
R1294	D-glucose exchange	[e]: D-glucose ⇔	−20	1000
R1196	Sulfate exchange	[e]: SO_4 ⇔	−1000	1000
R1204	O_2 exchange	[e]: O_2 ⇔	−1000	1000
R1211	Phosphate exchange	[e]: pi ⇔	−1000	1000
R1218	Ammonia exchange	[e]: NH_4 ⇔	−1000	1000
R1221	H_2O exchange	[e]: H_2o ⇔	−1000	1000
R1228	CO_2 exchange	[e]: CO_2 ⇔	−1000	1000
R1305	proton exchange	[e]: H ⇔	−1000	1000

[1] Model yli v1.7 and iYL619_PCP are equal in predicting the glucose minimal media; [2] LB indicates the lower bounds of reaction, unit is mmol/g DCW/h; [3] UB indicates the upper bounds of reaction, unit is mmol/g DCW/h.

Additionally, based on different specific glucose consumption rate, robustness analysis was performed to examine the prediction growth ability using model iYL619_PCP and model iYL619_PCP without R0437, respectively (Figure 2). The curve indicated the model without R0437 was more rational. In addition, model without R0437 also can rationalize metabolic flux distribution in both the EMP pathway and the pentose phosphate pathway (PPP), as computed with ^{13}C flux analysis [3].

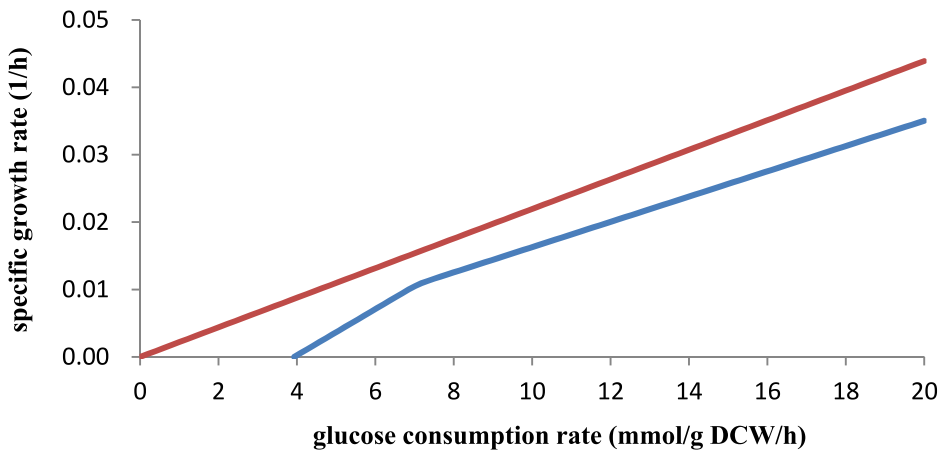

Figure 2. The robustness analysis results of glucose consumption to growth in model iYL619_PCP. Red and blue lines stand for the result of the model with R0437 and the model without R0437, respectively.

The updated genome-scale metabolic model of *Y. lipolytica* was renamed yli v1.7, which accounts for 594 genes, 1139 reactions and 844 metabolites. Subsequently, we used model yli v1.7 to predict the potential benefit of supplementing the minimal media with amino acids or several cofactors in terms of FPP production based on FBA metabolic flux distributions. And all simulations were acquired by using constraints-based flux balance analysis [4] with Cobra Toolbox 2.0 in Matlab R2011a.

Reference

1. Pan, P.; Hua, Q. Reconstruction and in silico analysis of metabolic network for an oleaginous yeast, *Yarrowia lipolytica*. *PLoS ONE* **2012**, *7*, e51535.
2. Nicaud, J.M. *Yarrowia lipolytica*. *Yeast* **2012**, *29*, 409–418.
3. Christen, S.; Sauer, U. Intracellular characterization of aerobic glucose metabolism in seven yeast species by 13C flux analysis and metabolomics. *FEMS Yeast Res.* **2011**, *11*, 263–272.
4. Orth, J.D.; Thiele, I.; Palsson, B.Ø. What is flux balance analysis? *Nat. Biotechnol.* **2010**, *28*, 245–248.
