# Supplementary material for: Flux Balance Analysis Inspired Bioprocess Upgrading for Lycopene Production by a Metabolically Engineered Strain of Yarrowia lipolytica
Source: Metabolites. 2015 Dec 21;5(4):794–813. doi: 10.3390/metabo5040794 (PMC4693195; doi:10.3390/metabo5040794)
Supplement: Supplementary File 1 [file metabolites-05-00794-s001.zip › metabolites-99322-supplementary-final/Supplementary File 3.pdf]

Supplementary File 3

Simulation Results

In model yli v1.7, R0763 is the only reaction, in which farnesyl diphosphate (FPP) is produced; R2000 is the reaction of growth (biomass formation reaction). Besides, the maximum flux of R2000 (specific growth rate) is 0.0350 h^{-1} in the glucose minimal media. The model yli v1.7 and iYL619_PCP predicted the equal glucose minimal media, and the composition of minimal media in experiment is as follows: glucose (20 g/L), NH_4SO_4 (3 g/L), KH_2PO_4 (2 g/L), in which the maximum specific growth rate is 0.0352 h^{-1} (Supplementary File 2, Figure 1), indicating that model yli v1.7 predicted the maximum specific growth rate more accurately.

The fluxes of exchange reactions of the updated model were generated through FBA. Exchange reactions with negative fluxes indicated compounds that needed to be consumed when targeting a maximized production of FPP and the addition of those components into the culture medium was of crucial requirement. The list of exchange reactions with negative fluxes and their fluxes when glucose, fructose or both carbohydrates were selected as carbon sources is summarized in Table S1. The most prevailing class of exchange reactions included exchange reactions involving amino acids such as L-isoleucine, L-leucine, L-valine, L-asparagine, L-histidine, L-methionine, L-tryptophan, guanine and L-lysine. The additional exchange reactions were those of O_2, thiamine diphosphate, succinate, phosphate, ammonia, 4-aminobutanoate, ergosterol, ethanolamine, D-fructose, urea, D-glucose, hypoxanthine and (R)-pantothenate. The number of negative exchange reactions and fluxes varied widely with the nature of the carbon source opted for. Putting aside the fact that FBA only give one possible random solution instead of looking at all the possibilities (like Flux Variability Analysis would do, for example), it seems that the only "non-zeros" values in this table are those corresponding to O_2 exchange, D-fructose exchange and D-glucose exchange. All other values from −8.05e-35 to −1.46e-13 are most probably rounding errors of the LP solver. And so, all those values could be considered as zeros. However, in order to predict the potential benefit of supplementing the minimal media with of above amino acids or several cofactors in terms of FPP production based on FBA metabolic flux distributions, we relaxed the exchange reactions of model yli v1.7. We first tested effects of 18 amino acids (only 18 amino acid can be utilized in model yli v1.7) on FPP production in a definite specific growth rate (90% of the maximum specific growth rate), and found that all the 18 amino acids facilitated FPP production in different degrees (Table S2). However, only 8 amino acids (L-isoleucine, L-leucine, L-valine, L-asparagine, L-histidine, L-methionine, L-lysine, L-tryptophan) were found as amino acids that could be supplemented to the minimal medium owing to the guarantee of C/N ratio more than 10.

Besides, we also respectively changed the lower bounds of 18 amino acid exchange reactions at −2 mmol/g DCW/h to test effects of the 18 amino acids on FPP production. The fluxes of the 18 exchange reactions of amino acids are presented in Table S3.

Table S1. Flux balance analysis prediction of chemical components with FPP set as objective target using glucose and fructose as carbon sources.

Reaction ID	Exchange Reaction	Flux		
		Glucose (−20)	Fructose (−20)	Glucose (−10) + Fructose (−10)
**R1202**	L-isoleucine exchange	−1.63e-28	0	−2.00e-33
**R1203**	L-leucine exchange	−2.10e-28	0	0
**R1204**	O_2 exchange	−32.5082	−32.5082	−32.5082
**R1205**	L-valine exchange	−4.78e-29	−4.10e-32	−4.91e-33
**R1207**	Thiamine diphosphate exchange	−6.28e-18	0	3.53e-15
**R1208**	Succinate exchange	−9.32e-26	0	−2.30e-29
**R1211**	Phosphate exchange	−1.46e-13	3.94e-14	8.05e-14
**R1218**	Ammonia exchange	−3.49e-14	−1.86e-14	−8.10e-15
**R1239**	4-Aminobutanoate exchange	−1.97e-14	0	0
**R1245**	L-Asparagine exchange	0	0	−2.45e-33
**R1251**	Ergosterol exchange	−1.05e-29	0	−8.05e-35
**R1255**	dTTP exchange	0	0	−3.64e-34
**R1258**	Ethanolamine exchange	−1.97e-27	0	−1.73e-29
**R1261**	D-Fructose exchange	0	−20	−10
**R1267**	Guanine exchange	0	0	−1.62e-31
**R1268**	L-histidine exchange	0	0	−1.41e-33
**R1273**	L-methionine exchange	−3.58e-27	0	−1.41e-33
**R1275**	L-lysine exchange	0	0	−3.18e-33
**R1278**	(R)-Pantothenate exchange	0	−1.09e-14	0
**R1281**	Sulfite exchange	0	−1.09e-14	−5.59e-32
**R1285**	L-tryptophan exchange	0	0	−4.54e-34
**R1287**	Urea exchange	−1.15e-28	0	−2.18e-33
**R1294**	D-glucose exchange	−20	0	−10
**R1296**	Hypoxanthine exchange	4.16e-15	−7.57e-15	−7.11e-15

Table S2. Fluxes (mmol/g DCW/h) of 18 exchange reactions of amino acids used to test the whole effect on FPP production (Specific production rate of FPP and specific growth rate were 2.4688 mmol/g DCW/h and 0.0315 h^{-1}, respectively).

ID	Reaction Name	LB	UB	Flux	Min	Max
R1202	L-isoleucine exchange	−2	1000	−0.0042	−2	−0.0042
R1203	L-leucine exchange	−2	1000	−0.0085	−0.0085	−0.0084
R1205	L-valine exchange	−2	1000	−0.0019	−0.0019	−0.0019
R1226	L-glutamate exchange	−2	1000	−2	−2	−2
R1241	L-alanine exchange	−2	1000	−2	−2	−2.0000
R1244	L-arginine exchange	−2	1000	−2	−2	−2
R1245	L-asparagine exchange	−2	1000	−0.0052	−0.0052	−0.0052
R1248	L-cysteine exchange	−2	1000	−2	−2	−2.0000
R1268	L-histidine exchange	−2	1000	−0.0030	−0.0030	−0.0030
R1273	L-methionine exchange	−2	1000	−0.0030	−0.0030	−0.0030
R1275	L-lysine exchange	−2	1000	−0.0067	−0.0067	−0.0067

Table S2. *Cont.*

ID	Reaction Name	LB	UB	Flux	Min	Max
R1280	L-serine exchange	−2	1000	−2	−2	−2
R1283	L-threonine exchange	−2	1000	−2	−2	−2
R1285	L-tryptophan exchange	−2	1000	−0.0010	−2	−0.0010
R1298	L-aspartate exchange	−2	1000	−2	−2	−2
R1300	L-phenylalanine exchange	−2	1000	−2	−2	−2.0000
R1301	L-proline exchange	−2	1000	−2	−2	−2
R1303	L-tyrosine exchange	−2	1000	−2	−2	−2.0000

Table S3. Fluxes (mmol/g DCW/h) of 18 exchange reactions of amino acids used to test individual effects on FPP production.

ID	Reaction Name	Biomass	FPP	Flux	Min	Max
R1202	L-isoleucine exchange	0.0315	0.4342	−0.0042	−2	−0.0042
R1203	L-leucine exchange	0.0315	0.4352	−0.0085	−0.0085	−0.0085
R1205	L-valine exchange	0.0315	0.4350	−0.0104	−0.0104	−0.0104
R1226	L-glutamate exchange	0.0315	0.4763	−2	−2	−2.0000
R1241	L-alanine exchange	0.0315	0.5871	−2	−2	−2.0000
R1244	L-arginine exchange	0.0315	0.5159	−2	−2	−2.0000
R1245	L-asparagine exchange	0.0315	0.4344	−0.0052	−0.00519	−0.0052
R1248	L-cysteine exchange	0.0315	0.5878	−2	−2	−2
R1268	L-histidine exchange	0.0315	0.4347	−0.0030	−0.00298	−0.0030
R1273	L-methionine exchange	0.0315	0.4352	−0.0072	−0.00723	−0.0072
R1275	L-lysine exchange	0.0315	0.4353	−0.0067	−0.00673	−0.0067
R1280	L-serine exchange	0.0315	0.6640	−2	−2	−2
R1283	L-threonine exchange	0.0315	0.7025	−2	−2	−2
R1285	L-tryptophan exchange	0.0315	0.4339	−0.0010	−2	−0.0010
R1298	L-aspartate exchange	0.0315	0.6640	−2	−2	−2
R1300	L-phenylalanine exchange	0.0315	0.6215	−2	−2	−2
R1301	L-proline exchange	0.0315	0.5150	−2	−2	−2.0000
R1303	L-tyrosine exchange	0.0315	0.5650	−1.6980	−1.6980	−1.6980

Based on the fluxes above, we found all amino acids facilitated production of FPP in different degrees. However, only 8 amino acids (L-isoleucine, L-leucine, L-valine, L-asparagine, L-histidine, L-methionine, L-lysine, L-tryptophan) were chosen as additions of media owing to guarantee of C/N ratio more than 10.

Additionally, we tested effects of 10 factors that play significant roles on FPP production. First, we changed the lower bounds of exchange reactions of factors at −2 mmol/g DCW/h to test the whole effect on FPP production. The specific production rate of FPP and specific growth rate were 0.4607 mmol/g DCW/h and 0.0315 h^{-1}, respectively. And fluxes of the 10 exchange reactions of factors showed in Table S4. Besides, we also respectively changed the lower bounds of 10 exchange reactions factors at −1 mmol/g DCW/h to test effects of the 10 factors on FPP production. And fluxes of the 10 exchange reactions of factors showed in Table S5.

Table S4. Fluxes (mmol/g DCW/h) of 10 exchange reactions of factors used to test the whole effect on FPP production. (the specific production rate of FPP and specific growth rate were 0.4607 mmol/g DCW/h and 0.0315 h^{-1}, respectively; the unit is mmol/g DCW/h).

ID	Reaction Name	Lb	Ub	Flux	Min	Max
R1207	Thiamine diphosphate exchange	−1	1000	0.0000	0.0000	0.0000
R1239	4 Aminobutanoate exchange	−1	1000	−1	−1	−1.0000
R1251	Ergosterol exchange	−1	1000	−0.0002	−0.0002	−0.0002
R1258	Ethanolamine exchange	−1	1000	−0.0769	−0.0769	−0.0769
R1267	Guanine exchange	−1	1000	−1	−1	−0.0026
R1278	(R)-Pantothenate exchange	−1	1000	0.0000	0.0000	0.0000
R1281	Sulfite exchange	−1	1000	0.0000	0.0000	0.0000
R1287	Urea exchange	−1	1000	0.0000	0.0000	0.0000
R1296	Hypoxanthine exchange	−1	1000	−0.0023	−0.0023	−0.0023
R1306	Pyridoxine exchange	−1	1000	0.0000	0.0000	0.0000

Table S5. Fluxes of the 10 exchange reactions of factors used to test individual effects on FPP production. (The unit is mmol/g DCW/h).

ID	Reaction Name	Biomass	FPP	Flux	Min	Max
R1207	Thiamine diphosphate exchange	0.0315	0.4332	0.0000	0.0000	0.0000
R1239	4 Aminobutanoate exchange	0.0315	0.4525	−1	−1	−1.0000
R1251	Ergosterol exchange	0.0315	0.4333	−0.0002	−0.0002	−0.0002
R1258	Ethanolamine exchange	0.0315	0.4392	−0.0769	−0.0769	−0.0769
R1267	Guanine exchange	0.0315	0.4345	−1	−1	−0.0026
R1278	(R)-Pantothenate exchange	0.0315	0.4332	0.0000	0.0000	0.0000
R1281	Sulfite exchange	0.0315	0.4332	0	0	0.0000
R1287	Urea exchange	0.0315	0.4332	0	0.0000	0.0000
R1296	Hypoxanthine exchange	0.0315	0.4353	−0.0049	−0.0049	−0.0049
R1306	Pyridoxine exchange	0.0315	0.4332	0	0	0

Based on the fluxes above, we found that few factors have a significant effect on FPP production, which are inconsistent with literatures. Furthermore, 10 factors were all chosen as supplements of media to verify their effects on FPP production.
