# Supplementary material for: Flux Balance Analysis Inspired Bioprocess Upgrading for Lycopene Production by a Metabolically Engineered Strain of Yarrowia lipolytica
Source: Metabolites. 2015 Dec 21;5(4):794–813. doi: 10.3390/metabo5040794 (PMC4693195; doi:10.3390/metabo5040794)
Supplement: Supplementary File 1 [file metabolites-05-00794-s001.zip › metabolites-99322-supplementary-final/Supplementary File 5.pdf]

Supplementary File 5

Table S1. Primers used in this study for qRT-PCR. F = forward, R = reverse.

Primer	Primer Sequence
ACT1-F	5′ TCCAGGCCGTCCTCTCCC 3′
ACT1-R	5′ GGCCAGCCATATCGAGTCG 3′
crtE-F	5′ ATGGTGAGTGGCAGTAAA 3′
crtE-R	5′ AGGTGATCGTCAATGGAT 3′
crtB-F	5′ CTCAGACCCGCTATGTCA 3′
crtB-R	5′ CATCATCAGACCCACCAC 3′
crtI-F	5′ GCACCATACCATCTGTTT 3′
crtI-R	5′ GTAGAGCGAGAAGTCATC 3′
YALI0B08536g-F	5LI0B08536g-FAGTCATC 3′35
YALI0B08536g-R	5LI0B08536g-RAGTCATC 3′35
YALI0B16038g-F	5LI0B16038g-FAGTCATC 3′-3L
YALI0B16038g-R	5LI0B16038g-RAGTCATC 3′-3L
YALI0B16126g-F	5LI0B16126g-FAGTCATC 3′
YALI0B16126g-R	5LI0B16126g-RAGTCATC 3′
YALI0C18755g-F	5LI0C18755g-FAGTCATC 3′-3L
YALI0C18755g-R	5LI0C18755g-RAGTCATC 3′-3L
YALI0C18799g-F	5LI0C18799g-FAGTCATC 3″
YALI0C18799g-R	5LI0C18799g-RAGTCATC 3″
YALI0D14762g-F	5LI0D14762g-FAGTCATC 3′GC-30
YALI0D14762g-R	5LI0D14762g-RAGTCATC 3′CG-30
YALI0D17050g-F	5LI0D17050g-FAGTCATC 3′35
YALI0D17050g-R	5LI0D17050g-RAGTCATC 3′
YALI0D17556g-F	5LI0D17556g-FAGTCATC 3′
YALI0D17556g-R	5LI0D17556g-FAGTCATC 3′
YALI0E04807g-F	5LI0E04807g-FAGTCATC 3′T-3I
YALI0E04807g-R	5LI0E04807g-RAGTCATC 3′A-3I
YALI0E05753g-F	5LI0E05753g-FAGTCATC 3′-3L
YALI0E05753g-R	5LI0E05753g-RAGTCATC 3′-3L
YALI0E06193g-F	5LI0E06193g-FAGTCATC 3′-3L
YALI0E06193g-R	56193g-R93g-FAGTCATC 3
YALI0E11099g-F	5LI0E11099g-FAGTCATC 3′GAGAC-31
YALI0E11099g-R	5LI0E11099g-RAGTCATC 3′CCTTT-31
YALI0F04015g-F	5LI0F04015g-FAGTCATC 3′35
YALI0F04015g-R	5LI0F04015g-RAGTCATC 3′
YALI0F05632g-F	5LI0F05632g-FAGTCATC 3′-3′
YALI0F05632g-R	5LI0F05632g-RAGTCATC 3′
YALI0F30481g-F	5LI0F30481g-FAGTCATC 3′-3′y
YALI0F30481g-R	5LI0F30481g-RAGTCATC 3′-3′
zwf-F	5f-FF30481g-RAGTCATC 3′-3′
zwf-R	5f-RF30481g-RAGTCATC 3′-3′
