# Supplementary figures and images for: Flux Balance Analysis Inspired Bioprocess Upgrading for Lycopene Production by a Metabolically Engineered Strain of Yarrowia lipolytica
Source: Metabolites. 2015 Dec 21;5(4):794–813. doi: 10.3390/metabo5040794 (PMC4693195; doi:10.3390/metabo5040794)

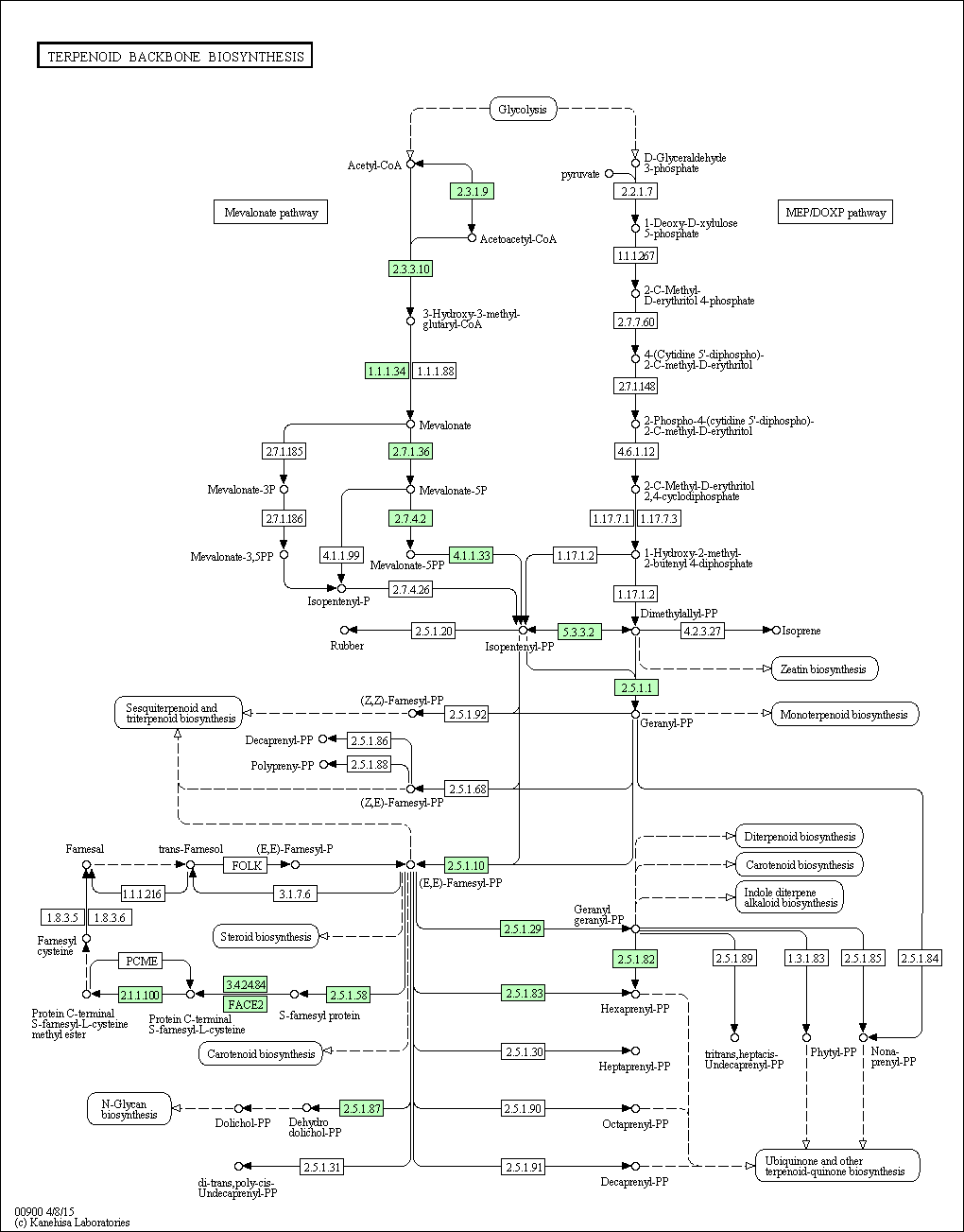

Supplement: Supplementary File 1 [file metabolites-05-00794-s001.zip › metabolites-99322-supplementary-final/Supplementary Figure S1.png]
